# hESC-derived striatal progenitors grafted into a Huntington’s disease rat model support long-term functional motor recovery by differentiating, self-organizing and connecting into the lesioned striatum

**DOI:** 10.1186/s13287-023-03422-4

**Published:** 2023-07-28

**Authors:** Roberta Schellino, Dario Besusso, Roberta Parolisi, Gabriela B. Gómez-González, Sveva Dallere, Linda Scaramuzza, Marta Ribodino, Ilaria Campus, Paola Conforti, Malin Parmar, Marina Boido, Elena Cattaneo, Annalisa Buffo

**Affiliations:** 1 Department of Neuroscience Rita Levi-Montalcini, University of Turin, 10126 Turin, Italy; 2 Neuroscience Institute Cavalieri Ottolenghi, University of Turin, 10043 Orbassano, Italy; 3 Department of Biosciences, University of Milan, 20122 Milan, Italy; 4 National Institute of Molecular Genetics “Romeo ed Enrica Invernizzi”, 20133 Milan, Italy; 5 Wallenberg Neuroscience Center and Lund Stem Cell Center, Lund University, 22184 Lund, Sweden

**Keywords:** Transplantation, Human embryonic stem cells, In vitro differentiation, Viral tracing, Medium spiny neurons, Direct and indirect pathways

## Abstract

**Background:**

Huntington’s disease (HD) is a motor and cognitive neurodegenerative disorder due to prominent loss of striatal medium spiny neurons (MSNs). Cell replacement using human embryonic stem cells (hESCs) derivatives may offer new therapeutic opportunities to replace degenerated neurons and repair damaged circuits.

**Methods:**

With the aim to develop effective cell replacement for HD, we assessed the long-term therapeutic value of hESC-derived striatal progenitors by grafting the cells into the striatum of a preclinical model of HD [i.e., adult immunodeficient rats in which the striatum was lesioned by monolateral injection of quinolinic acid (QA)]. We examined the survival, maturation, self-organization and integration of the graft as well as its impact on lesion-dependent motor alterations up to 6 months post-graft. Moreover, we tested whether exposing a cohort of QA-lesioned animals to environmental enrichment (EE) could improve graft integration and function.

**Results:**

Human striatal progenitors survived up to 6 months after transplantation and showed morphological and neurochemical features typical of human MSNs. Donor-derived interneurons were also detected. Grafts wired in both local and long-range striatal circuits, formed domains suggestive of distinct ganglionic eminence territories and displayed emerging striosome features. Moreover, over time grafts improved complex motor performances affected by QA. EE selectively increased cell differentiation into MSN phenotype and promoted host-to-graft connectivity. However, when combined to the graft, the EE paradigm used in this study was insufficient to produce an additive effect on task execution.

**Conclusions:**

The data support the long-term therapeutic potential of ESC-derived human striatal progenitor grafts for the replacement of degenerated striatal neurons in HD and suggest that EE can effectively accelerate the maturation and promote the integration of human striatal cells.

**Supplementary Information:**

The online version contains supplementary material available at 10.1186/s13287-023-03422-4.

## Background

Huntington’s disease (HD) is a monogenic dominant neurodegenerative condition caused by the expansion of the CAG trinucleotide repeat tract at the 5′ end of the Huntingtin (HTT) gene. Expansions larger than 36 CAG repeats trigger neuronal dysfunction and cell loss that preferentially affects the GABAergic projection neurons (medium spiny neurons, MSNs) in corpus striatum, leading to alteration of the cortico-basal ganglia circuitry, motor dyskinesias and progressive cognitive impairment [1].

While pharmacological treatment available for HD patients is only palliative, the development of HTT lowering strategies aimed at the attenuation of mutant HTT toxicity is promising but also challenging, as long-term efficacy and allele selectivity are yet to be demonstrated in a clinical setup [2–4]. Furthermore, these approaches need to account for the degree of neuronal loss at time of treatment, as this may reduce their effectiveness. New complementary cell therapies could mitigate dysfunctions due to neuronal death and circuit damage, as the reduced competences of degenerated cells can be potentially supplied by newly implanted cells.

Established technologies based on human embryonic stem cells (hESCs) offer the unprecedented possibility to generate large-scale neuronal identities in vitro through directed differentiation protocols [5], opening new avenues for cell replacement strategies. Because of the initial selective vulnerability of MSNs, HD may be particularly suitable for cell replacement approaches aimed at mitigating their loss. Recently, we used human MSN (hMSNs) progenitors obtained from ESCs [6] to test their short-time in vivo differentiation and therapeutic efficacy in a quinolinic acid (QA) lesioned rat model of HD [7]. At 2 months post-transplantation (MPT) a fraction of cells had matured toward authentic DARPP32/CTIP2/GABA expressing MSNs. Human striatal grafts showed a degree of integration into the host tissue—i.e., reached striatal targets and received synaptic inputs from local striatal afferents—with a partial rescue of lesion-induced sensorimotor deficits. However, long-range inputs to the grafts were not detected and, at that time point, the grafts did not promote recovery in complex motor tasks, nor there was evidence of maturation of the graft into a striatal-like tissue, such as the development of striosome-like features. Successful cell therapies require prolonged graft survival, and human neurons are known to complete maturation and synaptogenesis over months and years [8]. Consistently, only long-term observation may allow the full assessment and expression of the graft therapeutic potential. Hence, here we assessed the long-term molecular and functional properties of hESC-derived striatal progenitors prepared as described in Besusso et al. [7] and grafted into the striatum of QA-lesioned nude rats.

The influence of the environment and of the animal experience on neuronal structure, circuits and function has been demonstrated on striatal allografts in the rat chemical model of HD [9, 10]. However, it is not known whether nonspecific sensorimotor stimulation may have an impact on transplanted human striatal graft maturation, connectivity and functions. Thus, in this study, we also asked whether long-term exposure to an activity-enhancing enriched environment (EE) could improve human graft features.

Results show that long-term human grafts acquire a degree of striosome compartmentalization and reconstruct long-range connections, which is consistent with the evidence of a higher survival rate and a greater proportion of cells—compared to our short-term study [7]—that proceed toward maturation as striatal MSNs or interneurons and sustain lasting motor improvement. Acquisition of mature hMSN features and inputs from host neurons was further improved by exposure to EE.

## Methods

Part of the data used for specific analysis in this paper are from a previously published study [7] and are freely available on the Zenodo platform (link: https://zenodo.org/communities/nscr/?page=1&size=20). The qualification of the distinct cell preparations (Additional file 1: Fig. S1) was consistent between this and the former study. Thus, we compared the results of the new graft cohort with those of the previous one, despite the fact that our study does not represent a longitudinal assessment starting from the same cell preparation.

### Differentiation of hESCs and in vitro characterization

The human ESC line H9 (WA09—WiCell) was grown on Cultrex-coated (R&D systems) cell culture dishes (Eppendorf) and maintained in self-renewal using mTeSR medium (STEMCELL Technologies). When about 70% confluent, cells were passaged once a week using a solution containing 0.1 mM EDTA (Sigma-Aldrich) in a 1:10–12 proportion. Only cells with passages between 36 and 40 were used and screened for Mycoplasma contamination every month. Karyotype by means of q-Banding analysis (ISENET) was performed every 3 months to exclude for gross chromosomal abnormalities.

hESC H9 were then differentiated as described previously [7], following a modified version of the protocol used in Delli Carri et al. [6], aimed to enrich for non-proliferating committed striatal precursors. Briefly, cells were initially plated on Matrigel-coated dishes at 0.6 × 10^5^ cell/cm^2^ density with 10 μM ROCK inhibitor (Y-27632; Sigma), grown until reaching 70% confluence and differentiated upon exposure to induction medium based on DMEM/F12 (Thermo Fisher) supplemented with 1× N2 and 0.5× B27 without RA (Life Technologies), 10 μM SB431542 and 500 nM of LDN (provided both by Evotec). Medium was replaced every day. Starting from day in vitro (DIV) 5, cells were maintained for 3 weeks in a medium containing 200 ng/mL SHHC-25II (Tocris) and 100 ng/mL DKK1 (Peprotech), until DIV25. At DIV12, by Accutase (Millipore) treatment, the cells were lifted off in the presence of RI and then replated in Matrigel GFR (StemCell Technologies) at 2.5 × 10^3^ cell/cm^2^ density. Finally, H9 cells were cultured in a medium containing just 50 ng/mL BDNF with 1× N2 and 1× B27 for terminal differentiation.

As described in Besusso et al. [7], for synaptic tracing experiments, 15–18 DIV cells were transduced with a synapsin1-driven TVA-GP-GFP polycistronic lentiviral vector (Addgene no. 30195) with a MOI of 10 in the presence of 4 mg/mL polybrene (Sigma-Aldrich). The lentiviral construct carries the sequence for the expression of a synapsin promoter-controlled histone-tagged GFP, a TVA receptor (for the selective infection by the EnvA-pseudotyped ΔG-rabies virus, mRV, carrying mCherry reporter), and the glycoproteins driving mRV monosynaptic spreading [11]. This technique allows the identification of the infected starter cells targeted by mRV, characterized by nuclear GFP expression and mCherry cytoplasmic expression. Any neuron (traced cells) forming presynaptic contacts with the starter neurons will display mCherry positivity only, due to the selective transmission of mutated rabies virus retrogradely across active synapses.

For immunocytochemical analysis, cell cultures were fixed in 4% paraformaldehyde for 15 min at RT. After cell permeabilization with 5% normal goat serum (Vector) in PBS 0.1% Triton X-100 for 1 h at RT, cells were incubated overnight at 4 °C with primary antibodies. The next day, cells were incubated with the appropriate Alexa Fluor®-conjugated secondary antibodies (1:500; Life Technologies) and mixed with 0.1 μg/mL Hoechst (Invitrogen, cod. 33342) to counterstain nuclei. Images were acquired on a Leica TCS SP5 Confocal Laser Scanning Microscope (Leica Microsystems), using a 40× oil immersion objective (zoom = 1.7) or on a GE Healthcare IN Cell Analyzer 6000 (GE Healthcare Life Sciences), using a 40× objective. The percentage of cells positive for striatal markers DARPP32, CTIP2 and GAD67 was manually counted using the cell counter feature in ImageJ. For Ki67 and GSX2, the numbers of positive cells were counted using the free open-source CellProfiler 2.2.1 software.

At four different time points during differentiation (DIV0–DIV17–DIV20–DIV50), cells were collected and processed for total RNA extraction using TRIzolTM Reagent (Life Technologies). iScript cDNA Synthesis Kit (Bio-Rad) was used to retrotranscribed 500 ng of total RNA. Quantitative RT-PCR was performed using a CFX96TM Real-Time System (Bio-Rad) and analyzed with the CFX Manager Software (Bio-Rad). All reactions were performed in 15 uL containing 50 ng cDNA and SsoFastTM EvaGreen® Supermix (Bio-Rad) (Additional file 1: Fig. S1).

### Experimental model and surgical procedures

Athymic FOX-N1 adult rats (200–250 g, 7–8 weeks old) were purchased from ENVIGO Laboratories. The experimental procedures involving live animals were performed in strict accordance with the European directive (2010/63/EU), the Italian Law for Care and Use of Experimental Animals (DL116/92) and the University of Turin institutional guidelines on animal welfare, and authorized by the Italian Ministry of Health (Authorizations: 977-2016-PR and 327/2020-PR). Additionally, the ad hoc Ethical Committee of the University of Turin specifically approved this study. All studies involving animals are reported in accordance with the ARRIVE guidelines for reporting experiments involving animals.

Animals were deeply anesthetized by using 4% isoflurane (Isoflurane-Vet 100%, Merial Italy) vaporized in O_2_/N_2_O, 30%:70%. The rats were lesioned 8 days before transplantation by monolateral injection of 210 nmol of freshly made QA (1 μL total volume; Sigma-Aldrich) into the right striatum using the following stereotaxic coordinates (bregma): AP, + 0.6 mm; L, ± 2.8 mm; V, + 5.0 mm (Ugo Basile). At 20 DIV, after being detached with Accutase (STEMCELL Technologies) and 10 mM ROCK inhibitor (Sigma-Aldrich), hESC-derived striatal progenitors were resuspended at a concentration of 50,000 cells/mL, and 3 × 10^5^ cells (6 μL total volume) were grafted into the QA-lesioned striata using the following coordinates (bregma): AP, + 0.9 mm; L, + 3.1/− 3.1 mm; DV, 5.0 mm. Lesioned rats were coded and randomly assigned for cell transplantation or for a sham procedure. In sham rats, an equivalent volume of PBS was injected in the lesioned striatum. After the surgery, the head wounds were sutured using 4.0 surgical needles (Ethicon) and the animals were allowed to rest for the following days.

To perform mutated rabies-based monosynaptic tracing, 1 week before perfusion, under gaseous anesthesia (see above), the transplanted rats were injected with the EnvA-pseudotyped DGRV carrying mCherry reporter [11, 12]. For each animal, a dilution of 5% of mCherry RV, with a titer of 20–30 × 10^6^ TU/mL, was injected into the lesioned striatum (1 μL total volume) using the same stereotaxic coordinates used for the QA lesion.

### Behavioral procedures

#### Enriched environment

Housing conditions started 8 days post-striatal QA lesion, until the day of sacrifice 6 months post-lesion. Soon after cell transplantation, both sham and transplanted groups were divided into 2 experimental subgroups; animals with similar endurance behavior in the rotarod trials were distributed among the groups, so that each group started from average similar performances. One group of sham and grafted animals were housed in standard cages (up to 3/4 animals per cage), with water and food ad libitum and only nesting material in the cage; the second subgroups of sham and grafted athymic nude rats were housed (5 animals per cage) instead in large cages (100 × 95 × 54 cm), in which stimuli of different nature were placed (nesting materials, toys and wooden house, large plastic pipes, structures for climbing inside the cage), constituting the environmental enrichment that is known for encouraging exploration, social interaction and physical exercise [13]. The quality and disposition of those tools were renewed every 2 days to achieve higher levels of stimulation and associated physical activity. The floor of the arena was covered with fresh sawdust and allowed free access to food and water (adapted from [14, 15]).

#### Rotarod test

The rotarod test started 1 week before monolateral QA lesion to set up the baseline performance for each animal. Thereafter, rats were tested 1 week post-lesion/before graft, 2 weeks post-transplant and every month after transplantation. The behavioral test was conducted blind to the rat treatment(s). The animals were placed on a rotating rod (Ugo Basile) and a steady acceleration was applied from 4 to 40 rpm, setting 300 s as a maximum time [7, 16]. The test was repeated three times in each session and the latency to fall from the rotating rod was recorded, the average latency provided a measurement of rat motor coordination. Results from individual animals were normalized over the performance after lesion and expressed as mean.

Animals that did not show an impairment in the use of the contralateral paw soon after the lesion, those that did not complete the test up to the last time point (6 MPT), or in which the graft was not visible in histological samples were excluded from analysis.

### Brain histology and immunohistochemistry

Six months after transplantation, under deep general anesthesia (Isoflurane-Vet 100%), rats were transcardially perfused with phosphate buffer (0.1 M PB, pH 7.4), followed by cold 4% paraformaldehyde (PFA) in 0.1 M PB (pH 7.4). The brains were immediately dissected, postfixed for 2 h at 4 °C and cryoprotected in 30% sucrose (Sigma-Aldrich) in 0.1 M PB. The brains were then embedded, frozen in cryostat medium (Killik, Bio-Optica, Milan, Italy), and cryostat sectioned into free-floating, 40 μm thick, coronal sections that were stored in an antifreeze solution (30% ethylene glycol, 30% glycerol, 10% PB; 189 mM NaH_2_PO_4_; 192.5 mM NaOH; pH 7.4) at − 20 °C until being used.

Sections were then permeabilized with 0.5% Triton X-100 (Sigma-Aldrich) and blocked with 2% donkey serum (Sigma-Aldrich) for 1 h at RT, and then incubated overnight at 4 °C with the primary antibodies at the dilutions indicated in Additional file 1: Table S2. Sections were then exposed for 2 h at RT to secondary species-specific antibodies conjugated with Alexa Fluor dyes (Jackson Immunoresearch) and DAPI (Thermo Fisher Scientific). Slides were coverslipped with Mowiol (Millipore, Burlington, MA, USA). All images were collected using a Nikon Eclipse 90i (Nikon, Melville, NY) confocal microscope, or using a Leica TCS SP5 (Leica Microsystems, Wetzlar, Germany) confocal microscope. Fluorescent tile-scan images of entire brain slices were acquired using the Zeiss Axioscan 7 Microscope Slide Scanner (Zeiss). Adobe Photoshop 6.0 (Adobe Systems) was used to adjust image contrast, and Inkscape software (Free vector graphics editors) was used to assemble the final plates.

### Muscle histochemistry

For hematoxylin/eosin (H/E) staining, sections of fresh triceps and gastrocnemius muscles were sliced using the cryostat (Leica) in cross section at the thickness of 30 μm, and directly mounted on 4% gelatin-coated slides and air-dried overnight. Sections were then hydrated in distilled water, and stained firstly with hematoxylin, then with eosin (Bio-Optica), dehydrated in ascending series of ethanol (95–100%) and cleared in xylene (98.5%). Finally, sections were cover-slipped with Eukitt (Bio-Optica). The sections were drawn and analyzed by Neurolucida software (MicroBrightField Inc., Williston, VT, USA), and data were obtained by the associated data analysis software Neurolucida Explorer (MicroBrightField).

### Quantitative analyses

The analyses were conducted blind to the rat treatment(s).

#### Analysis of graft volume

Graft volume quantification was performed as in Besusso et al. [7]. Briefly, to estimate the graft volumes, the sections were mapped using Neurolucida Software and analyzed with Neurolucida Explorer (MBF Bioscience). The graft area was measured on sections showing Human Nuclei-positive (HuNu^+^) staining and were 200 µm apart from each other. From these measurements, the volumes of the grafts were calculated using Cavalieri’s principle. In both SE and EE conditions, grafts often expanded to include the globus pallidus.

#### Analysis of marker expression

The positivity to all the markers analyzed was quantified by counting the percentage of labeled cells over the total HuNu^+^ cell population, using confocal images (40× magnification, 1 µm *z*-step size, 10 µm *z*-volume, acquisition speed 100 Hz, format 1024 × 1024 pixels) of the striatal region. For each animal, three/four sections of the striatum were processed for quantifications, and at least three/four images were acquired for each section.

#### Analysis of starter and traced cell number and spatial distribution

In the viral-tracing analysis, starter (mCherry^+^/GFP^+^) and traced (mCherry^+^/GFP^−^) cells were counted at confocal microscope in at least 12 slices/animal containing the striatum, along the brain antero-posterior axis, and the average number of starter or traced cells per slice was determined as in Besusso et al. [7]. GraphPad outlier calculator, using alpha = 0.05 for significance level, confirmed the absence of outliers in these data. The expression of selected markers was evaluated by counting the percentage of labeled cells on the mCherry-positive elements (GFP^+^ or GFP^−^). The co-localization with GFP and the other cellular markers was assessed to identify mCherry-positive cell identity (starter or traced), and the expression of the selected marker was represented as the percentage of marker positive cells over the entire labeled mCherry^+^/GFP population.

For analysis of traced cells at extrastriatal sites and distribution analysis along the anterior–posterior brain axis, at least 12 tile-scan images (3–4 sets of 4 consecutive slices with a step-size of 1200 μm between sets) were acquired using Axioscan (Zeiss). Images were processed and analyzed using ImageJ (NIH). Neurons were assigned to specific brain regions based on classifications of the Rat Brain Atlas [17], using anatomical point of references in the sections visualized by DAPI. Multichannel immunofluorescence was performed to identify neuronal population and identity (traced or starter cells), to confirm that mCherry^+^ cells at extrastriatal sites were cells from the host (HuNu^−^).

In some instances, the distribution of human cells positive for a specific marker in adjacent sections was mapped over a schematic representation of rat brain coronal sections, after drawing the graft contours by Inkscape software, on entire slice images acquired by Axioscan (Zeiss). Briefly, after the isolation of the specific channel for a marker on ImageJ, a threshold was applied to segment the image. Then, the segmented image was imported on Inkscape software and converted into a vector bitmap image. The vector image was then overlapped with the Axioscan image, and the oversaturated signals were then removed with Inkscape software.

#### Morphometric analysis of mCherry^+^ graft cells

Images of isolated HuNu^+^/mCherry^+^ neurons encompassing the entire nucleus of the cells (*z* stack of 20–25 μm, Fig. 3A) were acquired using a confocal microscope (images: 40× magnification, 1 µm *z*-step size, acquisition speed 100 Hz, format 1024 × 1024 pixels) and cells were categorized in spiny or aspiny neurons according to the presence or absence of dendritic spines (Additional file 1: Table S1 and Additional file 1: Fig. S4A). A further subsetting was applied to spiny neurons in order to distinguish neurons defined in Graveland et al. [18] as bona fide hMSNs. To this aim, a spine density cutoff of 3.6 spines/10 um dendrite was applied to measurements on grafted cells. Such cutoff value was identified as the intersection of the normal spine density distributions (probability distribution function) obtained from means and SD values reported in Graveland et al. [18] (Additional file 1: Fig. S4B).

To measure spine densities, for a subset of cells confocal *z*-stack images of 27 ± 3 μm were acquired at higher magnification (objective: 63×, digital zoom: 3.40, gain: 50%, resolution: 1024 × 1024, speed: 100 Hz, *z*-step size: 0.5 μm). The mCherry signal was digitally processed with the ImageJ-Fiji software (enhance contrast: 0.3%, unsharp mask: 0.6 px; multi-point tool). Spine counting was performed along primary and secondary dendrites up to 20 μm distance starting from the first spine observed close to the cell body (54 and 68 dendrites, from 4 animals per group, SE and EE, respectively). Dendritic spines were defined as protrusion of the neuronal dendrite with any of the following morphology: mushroom, filopodia, thin and stubby. To avoid an overestimation, the stubby shape was considered in the counting only when the protrusion was evident with respect to the irregular surface of the dendrites, meaning that it formed a 90°/80° angle with the protrusion top. The dendrite extent was manually defined by drawing the process through the *Z*-stack, starting from the soma. The former results are presented in a range of 10 μm following Graveland et al. [18].

#### Morphometric analyses of DARPP32^+^ graft neurons and muscle fibers

Grafted cell body area and Feret’s diameter were measured on HuNu/DARPP32-positive cells, on confocal images using ImageJ software. 30 human striatal cells per animal per group were analyzed, and the average values calculated. The distance between HuNu^+^ grafted cells was measured by the nearest neighbor distance calculation on ImageJ. About 150 measurements were evaluated for each group.

After H/E staining, gastrocnemius and triceps muscles were morphologically evaluated in terms of mean fiber area and Feret’s diameter, by drawing the section with Neurolucida software (MicroBrightField Inc., Williston, VT, USA), and data were obtained by the associated data analysis software NeuroExplorer (MicroBrightField). More than 100 fibers were drawn and analyzed for each animal of each group (sham and transplanted animals maintained in SE or EE); we then averaged the means obtained from a single animal. Both contralesional and ipsilesional muscles were analyzed.

#### Marker area positivity

For the analysis of vGlut-1/2, TH and IBA1 labeling, the positive fractioned area in the field of view (i.e., the percentage of positive pixels throughout the entire image) was quantified by ImageJ on confocal images (40× magnification, 1 µm *z*-step size, 10 µm *z*-volume, acquisition speed 100 Hz, format 1024 × 1024 pixels; twelve fields for each animal of the healthy and lesioned striatum), after automatically applying a Gaussian blur to each picture, and setting a local intensity threshold of a defined radius around each pixel (following Besusso et al. [7]).

The positive fractioned area occupied by graft-derived STEM121^+^ fibers in the striatal target regions of the brain was manually traced and quantified over the nucleus area traced (extension of fibers over μm^2^) using Neurolucida and Neurolucida Explorer software (MBF Bioscience) in a blinded fashion. The size of DARPP32-positive patches in the striata and the number of DARPP32-positive cells in the patches were analyzed using Neurolucida and Neurolucida Explorer softwares (MBF Bioscience).

#### BDNF expression levels

The analysis of BDNF staining levels was performed according to Fucà et al. [19], with modifications. Briefly, single 1 μm-focal plane confocal images were collected under a 40× objective. On such images we measured the mean brightness intensity of 100 BDNF^+^ cells/area/animals by using ImageJ. The background brightness, taken from a non-stained region of the same focal plane, was subtracted from the brightness measurements for each image. Each measure was then assigned to one of three categories of staining intensity, ranging from the lowest to the highest value of BDNF intensity detected: weak = 0–33%, medium = 34–66%, strong = 67–100% of maximum staining intensity. 10 cells per image/10 images per animal (100 cells, in total) were measured for each animal.

### Statistical analysis

The data were represented as means ± standard error of mean (SEM). In the scatter plots, each dot represents individual values for the specific data. Statistical analyses were performed by GraphPad Prism 8.0 software (GraphPad Software, San Diego, CA, USA). The Kolmogorov–Smirnov test was first applied to test for a normal distribution of the data and, accordingly, appropriate tests were applied. Data were analyzed using two-tailed unpaired Student’s *t* test (with normal distribution), Mann–Whitney U (with not normally distributed data), one way and two-way ANOVA and Kruskal–Wallis test, respectively, followed by Bonferroni/Sidak test or Dunn’s multiple comparison test for post hoc analyses. Using analysis of variance tests, the between-subjects factor of Group and within-subjects factor of Time were used. In the rotarod analysis, to obtain an estimate of the performance, we performed a linear regression on latency to fall values for each experimental group. We used Chi-square test to compare the distribution of frequencies of staining intensity categories and cell type classes. Results were deemed to be statistically significant when *p* was < 0.05; * *p* < 0.01; ** *p* < 0.001; ***. All details of statistical analyses are reported in Additional file 1: Table S1.

## Results

### Transplanted human striatal progenitors show long-term survival and progressive differentiation

Upon confirmation of acquisition of human ventral telencephalic identity by the cultured cells, as judged by the progressive and consistent expression of subpallium and lateral ganglionic eminence (LGE) specific markers (Additional file 1: Fig. S1), human striatal progenitors were harvested at 20 DIV and grafted into the QA-lesioned striatum of immunocompromised rats (3 × 10^5^ cells per animal). Rats were then housed in standard (SE) or enriched (EE) environments for 6MPT. Based on the similarities between current and former transplantation work [7], in vivo results obtained in SE housing were therefore compared to formerly published data obtained at 2MPT [7] and then confronted with those collected in EE housing.

**Fig. 1:**
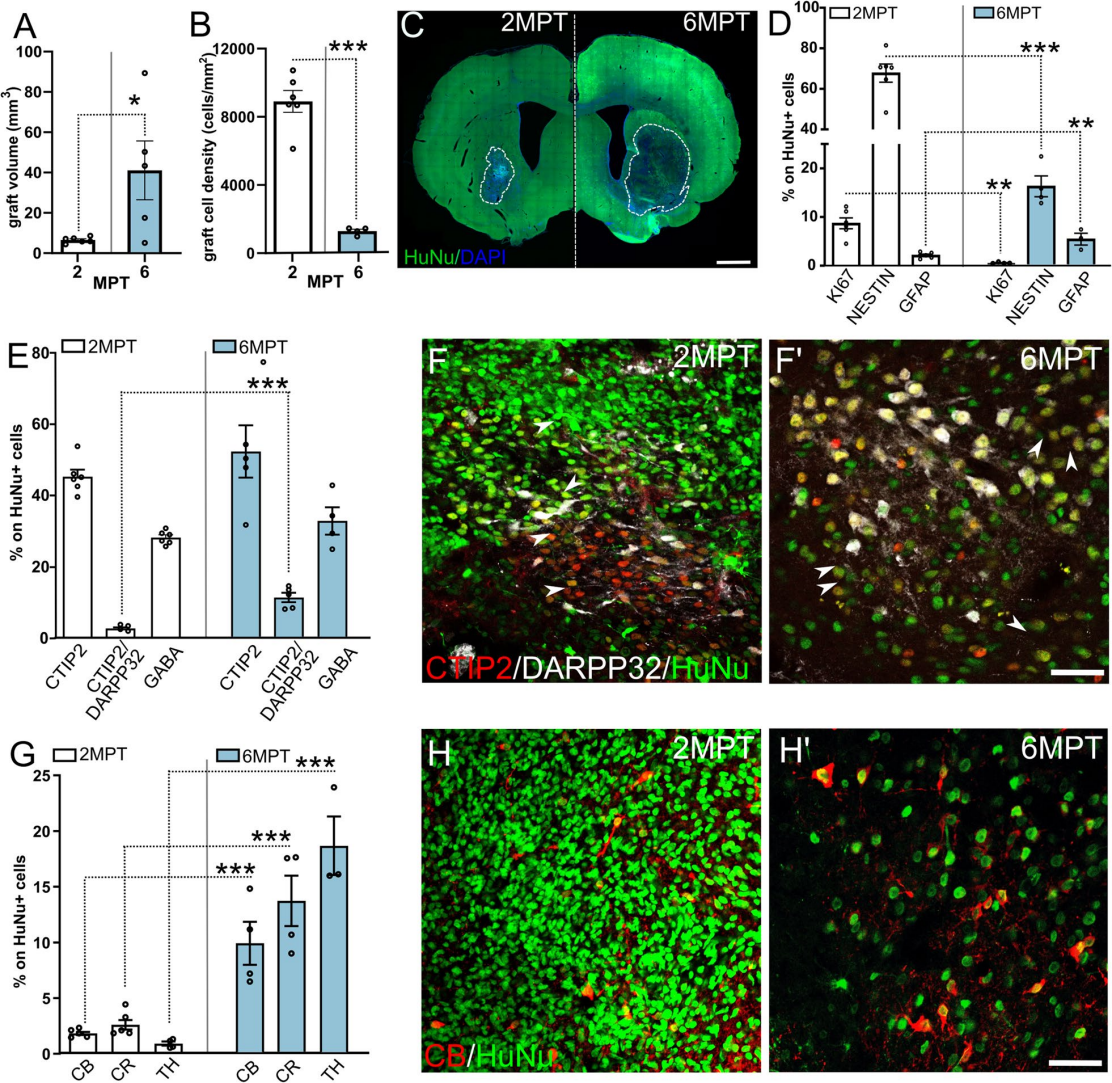
Analysis of human striatal grafts at short and long transplantation times in standard housing. **A** Differences in graft volume between 2 and 6MPT (unpaired *t* test; *p* < 0.05; *N* = 6 animals 2MPT; 5 6MPT SE). **B** Comparison of graft cell density at 2MPT and 6MPT (unpaired *t* test *p* < 0.001; *N* = 6 animals 2MPT; 4 6MPT SE). **C** Images depict the grafted hemisphere at 2MPT and 6MPT showing graft size and position (dotted lines) at 2MPT or 6MPT. **D** Proportions of HuNu^+^ cells expressing the proliferative marker Ki67 (unpaired *t* test, *p* < 0.01; *N* = 6 2MPT; 4 6MPT), or the immature marker NESTIN (unpaired t test, *p* < 0.01; *N* = 6 2MPT; 4 6MPT), or the astrocyte marker GFAP (unpaired *t* test, *p* < 0.01; *N* = 6 2MPT, 3 6MPT) at 2 or 6 MPT in HuNu^+^ cells (*N* = 6 animals 2MPT; 5 6MPT SE). **E** Proportions of striatal marker expression in HuNu^+^ cells at 2 and 6 MPT (unpaired *t* test, 2MPT vs. 6MPT SE; CTIP2, *p* > 0.05 ns; DARPP32, *p* < 0.001; CTIP2/DARPP32, *p* < 0.001; GABA, *p* > 0.05 ns; *N* = 6 animals 2MPT; 5 6MPT SE). **F** and **F′** Progressive maturation of hESC-derived striatal progenitors (HuNu^+^) into MSN neurons expressing CTIP2 (in red) and DARPP32 (in white). Arrowheads indicate some HuNu^+^/CTIP2^+^ neurons. **G** Proportions of interneuron markers expressed by HuNu^+^ neurons at 2 and 6 MPT (unpaired *t* tests, *p* < 0.001; CB and CR, *N* = 6 2MPT; 4 6MPT SE; TH, *N* = 4 2MPT, 3 6MPT). **H** and **H′** Representative graft images showing a fraction of grafted cells (in green) positive for calbindin (CB, in red) at 2 and 6 MPT. Data are represented as mean ± SEM. Scale bars: 1 mm (**C**); 50 μm (**F**–**H′**)

Grafted human striatal progenitors survived well long-term after transplantation in SE QA-lesioned nude rats, resulting in 73.4% of transplanted animals with a clear graft at 6MPT. Compared to 2MPT, at 6MPT grafts showed an increased size (about sevenfold), although with a degree of variability (Fig. 1A and Additional file 1: Table S1). Yet, the estimated fraction of engrafted HuNu^+^ cells over the inoculated cells remained similar to that at 2MPT (*p* > 0.05; Additional file 1: Table S1), accounting for about ¾ of the grafted cells, and corresponding to a decreased cell areal density at late versus early time points (Fig. 1B, C and Additional file 1: Table S1). Accordingly, by nearest neighbor distance analysis, at 6MPT we found increased distances between HuNu^+^ nuclei compared to 2MPT (Additional file 1: Fig. S2A–B′) and observed numerous host (HuNu^−^) astrocytes and microglial cells infiltrating the graft (Additional file 1: Fig. S2C–C′). Further, grafted human cells also almost doubled their size, as shown by cell body size measurements (*p* < 0.001; Additional file 1: Fig. S2D, E and Additional file 1: Table S1). Overall, these data suggest a progressive growth in size of engrafted human striatal cells and expansion of the neuropil, which become infiltrated by host glial cells during graft maturation.

We then looked at the differentiation of grafted cells at 6MPT. We found that cell proliferation and expression of the neural progenitor marker NESTIN were negligible or greatly reduced compared to 2MPT (Fig. 1D and Additional file 1: Table S1). In parallel, the proportions of cells displaying mature striatal neuronal markers increased. Similarly to data at 2MPT, the bona fide MSN lineage marker CTIP2 was found in about half of HuNu^+^ cells, while human cells double positive for CTIP2 and DARPP32 expression increased about fourfold, accounting for 11% of all grafted cells (Fig. 1E–F′ and Additional file 1: Table S1), showing a progression of hMSN progenitors toward maturation. Furthermore, 30% of graft-derived human cells expressed GABA (Fig. 1E and Additional file 1: Table S1), in line with the acquisition of an inhibitory cell identity, as expected for striatal neurons. Also, the fractions of striatal interneurons [Calbindin (CB)^+^, Calretinin (CR)^+^, Tyrosine Hydroxylase (TH)^+^] expanded about tenfold, compared to 2MPT data (Fig. 1G–H′ and Additional file 1: Table S1). Of note, TH^+^ cells showed GABA coexpression supporting an interneuronal rather than dopaminergic identity [20]. Additional file 1: Fig. S2H–H′–H″). Moreover, a small graft fraction (≈ 5%) was composed by GFAP+ astrocytes, which doubled compared to short-term grafts (Fig. 1D and Additional file 1: Table S1). In order to monitor the occurrence of non-striatally specified cell types, we assessed the expression of markers of cortical neurons which are not expressed in the mature rodent striatum [21]. In line with the in vitro differentiation data [7, 22], only rare HuNu^+^ grafted cells exhibited non-striatal identity, as shown by the expression of the cortical markers SATB2 or TBR1 [21], Additional file 1: Fig. S2F, G). Overall, these data show that human striatal grafts have proceeded in their differentiation during 6MPT and are formed by a composite of cell types including maturing and differentiated MSNs, interneurons and astrocytes.

### Enriched environment promotes the maturation of grafted hMSN progenitors

**Fig. 2:**
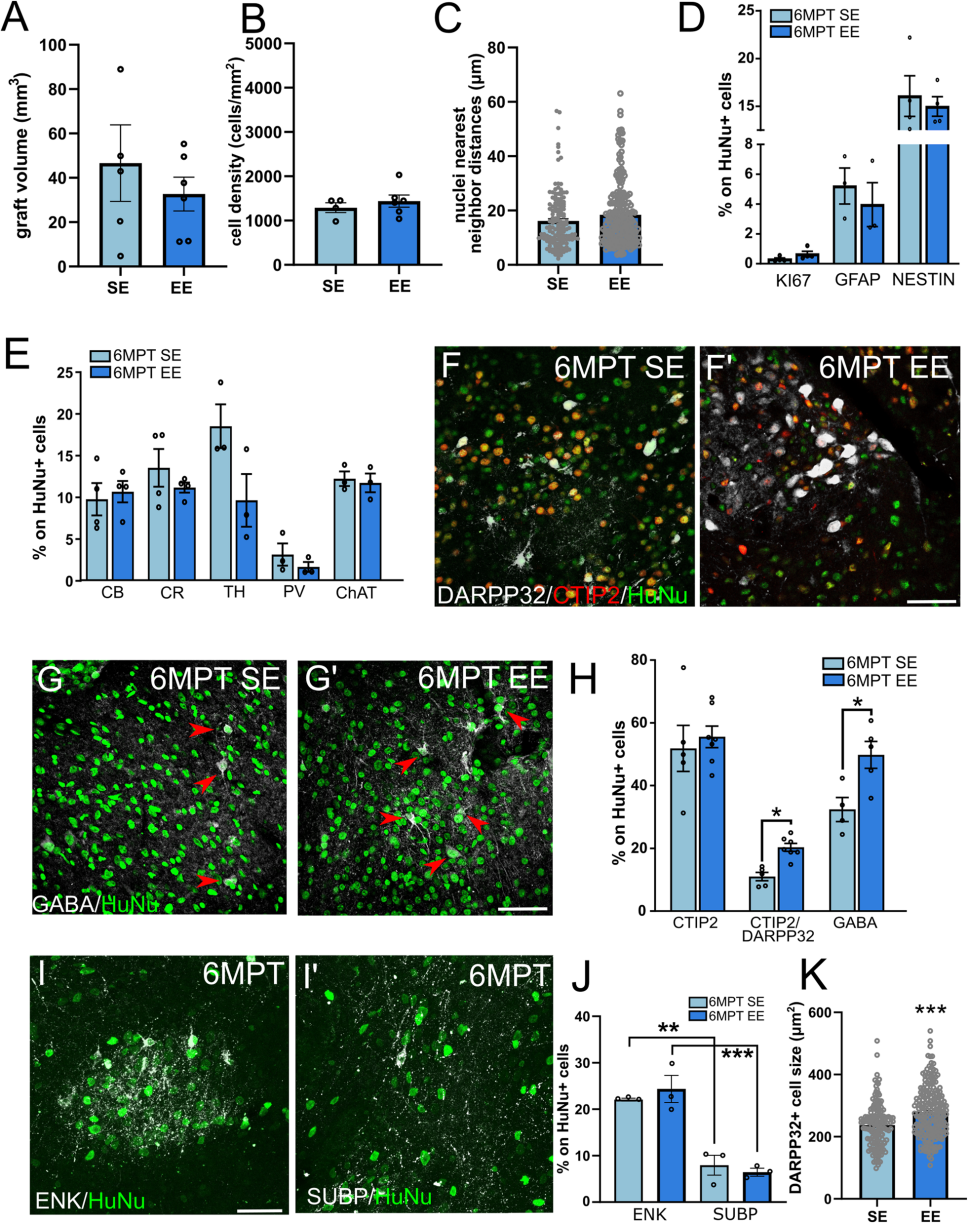
Analysis of human striatal grafts in standard and environmental enrichment housing at 6 MPT. **A** Similar graft volumes in SE and EE conditions (unpaired *t* test, *p* > 0.05 ns. *N* = 5 animals SE, 6 EE). **B** Similar graft cell densities in SE and EE conditions (unpaired *t* test, *p* > 0.05 ns. *N* = 4 animals SE, 6 EE). **C** Similar nearest neighbor distances of human nuclei in SE and EE groups (unpaired *t* test, *p* > 0.05 ns; *N* = 30 cells/animal; 5 animals SE; 6 EE). **D** Proportions of proliferative (KI67+; *N* = 4 SE; 4 EE), astroglial (GFAP^+^; *N* = 3 SE; 3 EE), or immature (NESTIN^+^; *N* = 4 SE; 4 EE) cells do not differ between SE and EE conditions (unpaired *t* tests, *p* > 0.05). **E** Proportions of interneuron markers expressed by grafted cells in SE and EE conditions (unpaired *t* tests, *p* > 0.05 ns; CB and CR, *N* = 4 SE, 4 EE; TH, PV and ChAT, *N* = 3 SE, 3 EE). **F** and **F′**) Representative images showing increased DARPP32 expression in CTIP2^+^ neurons in EE housing compared to SE. **G** and **G′** GABA-positive (red arrows) neurons in SE and EE human grafts. **H** Increased expression of DARPP32 and GABA in HuNu^+^ neurons in EE compared to SE (CTIP2, unpaired *t* test, *p* > 0.05 ns; DARPP32 and CTIP2/DARPP32, unpaired *t* test, *p* < 0.05. *N* = 6 SE, 7 EE; GABA, unpaired *t* test, *p* < 0.05; *N* = 4 SE, 5 EE) **I** Enkephalin (ENK)-positivity (white) in HuNu^+^ graft cells (green). **I′** SubstantiaP (SUBP)-positivity (white) in HuNu^+^ graft cells (green). **J** Quantification of ENK and SUBP expression in human graft cells in SE and EE groups (One-way ANOVA, Bonferroni post hoc test, 6MPT SE ENK versus 6MPT SE SUBP, *p* < 0.01; 6MPT EE ENK vs. 6MPT EE SUBP, *p* < 0.001. *N* = 3 SE, 3 EE). **K** Analysis of DARPP32-positive human MSN size (unpaired *t* test, *p* < 0.001. *N* = 30 cells/animal; 5 animals SE, 7 EE). Data are represented as mean ± SEM. Scale bars: 50 μm

Exposure to EE is known to modify brain circuits and to affect the properties of striatal allograft transplanted into the adult lesioned rat brain [10, 23, 24]. To assess if also grafts of human cells respond to EE stimuli, a group of transplanted QA-lesioned rats was maintained in EE housing from the time of cell implantation until sacrifice. Compared to animals housed in SE, exposure to EE did not significantly affect graft size or density. Indeed, the fractions of engrafted cells, average graft volume, cell density and nuclear nearest neighbor distances were all comparable to the SE condition (Fig. 2A–C and Additional file 1: Table S1).

Also, in comparison with SE, HuNu^+^ grafted cell proliferation and immaturity traits were not influenced by EE exposure (Additional file 1: Table S1), nor was the extent of differentiation along astroglial (GFAP^+^) and interneuronal (CB^+^, CR^+^, TH^+^) lineages (*p* > 0.05) (Fig. 2D, E and Additional file 1: Table S1). However, EE did exert a selective effect on the maturation of cells expressing typical markers of hMSNs. In particular, while the proportion of CTIP2 expressing cells did not vary with respect to SE (Additional file 1: Table S1), the fraction of DARPP32^+^/CTIP2^+^ human cells doubled compared to SE (*p* = 0.027) (Fig. 2F–H and Additional file 1: Table S1). Moreover, GABA expression also significantly increased within the EE stimulated grafts, accounting now for about 50% of grafted cells (vs. about 30% of expression in SE) (Fig. 2G, H and Additional file 1: Table S1).

To shed further light on the subtype(s) of MSNs in the grafts, we investigated the expression of neuropeptides commonly associated with striatal direct or indirect pathways [25], namely Substance P (SUBP) and Enkephalin (ENK), respectively. Both markers were expressed by HuNu^+^ cells at 6MPT in SE, with a significant predominance of neurons of the indirect pathway compared with the direct one (Fig. 2I, J). ENK expression in EE grafts was similar to that in SE and dominated over SUBP (more than 20% of human cells expressing ENK compared to about 7% of SUBP^+^/HuNu^+^ cells in both conditions) confirming a prevalence of indirect pathway neurons (Fig. 2I, J, and Additional file 1: Table S1). In line with these data, we also observed several grafted neurons positive for the dopamine receptor D2, which neurochemically defines the indirect pathway, while direct pathway human cells labeled by anti-D1 receptor antisera were rare and/or below the detection limit of the antibody (Additional file 1: Fig. S3A–C; antibody specificity was confirmed by staining in the healthy contralateral striatum: Additional file 1: Fig. S3D–d″). Finally, in agreement with former data on striatal allografts [10], the area of DARPP32^+^/HuNu^+^ somata significantly increased of about 17% in the animals housed in EE versus SE (Fig. 2K and Additional file 1: Table S1), suggesting incremented neuronal metabolism and activity [24]. Overall, these results show that EE promotes the acquisition of a more mature MSN neurochemical profile in human striatal grafts.

### Grafted human neurons display morphometric features of adult human MSNs and appear more integrated in EE

**Fig. 3:**
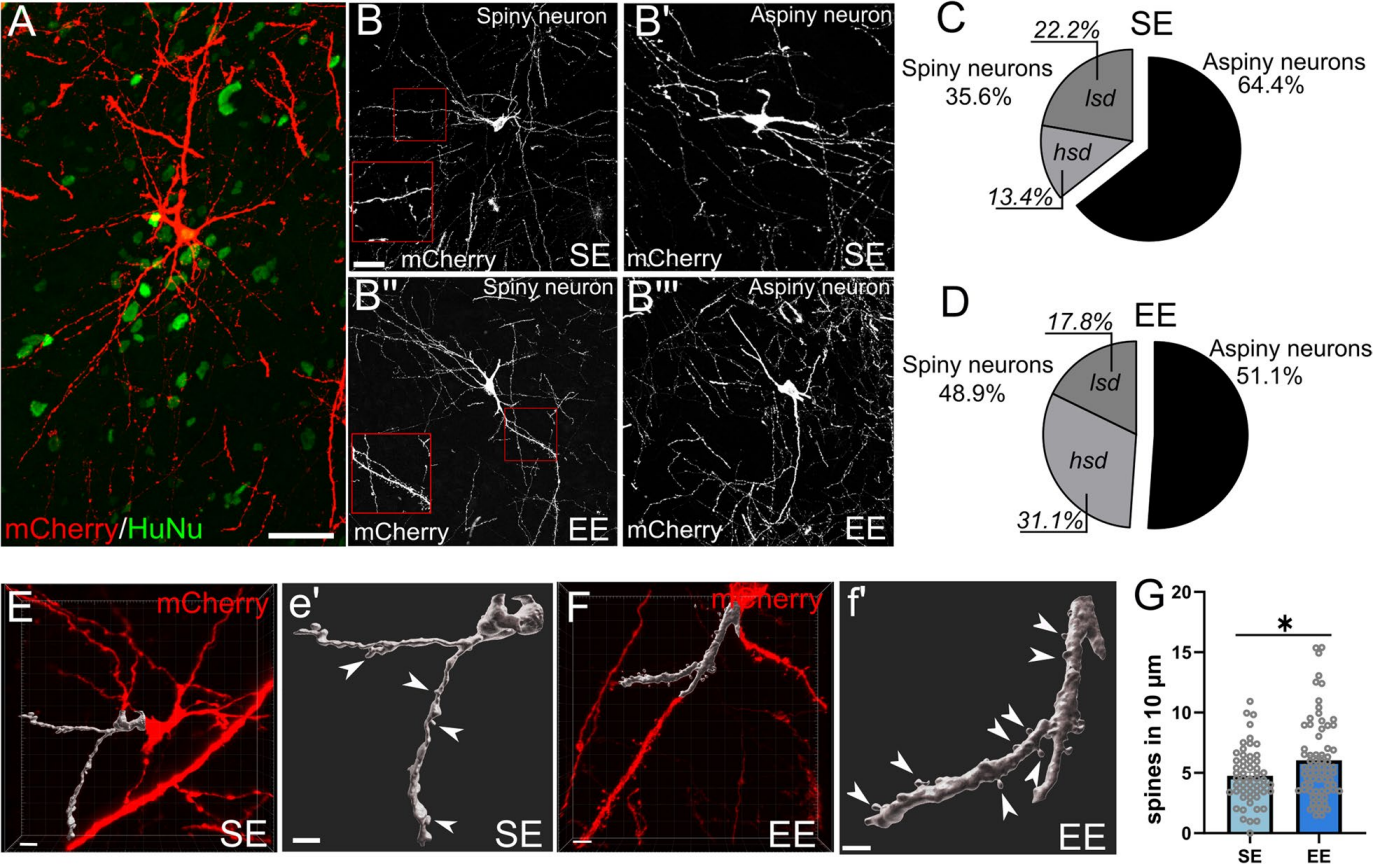
Morphological analysis of hMSNs reveals enhanced differentiation in environmental enrichment compared to standard conditions. **A**-**B′″** Representative images of human (HuNu^+^) neurons expressing mCherry upon transduction with mRV showing Spiny or Aspiny features in SE (**B**, **B′**) and EE (**B″**, **B′″**). **C** and **D** Proportions of Spiny and Aspiny human neurons in SE and EE conditions. **E**, **e′** and **F**, **f′** 3D rendering of mCherry expressing neurons in SE or EE displaying representative examples of dendrites (gray segment) and spines (arrowheads) considered for analysis (Chi-square test Aspiny vs. Spiny SE vs. EE, *p* = 0.230; *N* = 6 cells SE, 14 EE; chi-square test *hsd neurons* vs. others SE vs. EE, *p* = 0.042. *N* = 45 cells SE; 45 EE). **G** Spine number along 10um dendrites in SE and EE groups (one-tailed Mann–Whitney test, *p* = 0.028. SE *N* = 54 dendrites; EE, *N* = 68 dendrites). Scale bars: 50 μm (**A**, **B**); 3 μm (**E**, **e′**, **F**, **f′**)

As an additional approach to investigate the maturation and striatal identity of the grafted cells, we performed morphometric analyses on human grafted neurons expressing the mCherry reporter of mutated rabies virus (mRV, see “Methods” section and below for details; Fig. 3A). We focused on dendritic spines of multipolar neurons (with soma size in the range indicated in Graveland et al. [18], see Additional file 1: Fig. S4) because spine development correlates well with neuronal maturation and functional integration [8] and, above all, high spine density is a key morphological trait of mature MSNs. In the inspected human transplanted neurons exposed to SE, we found a relevant proportion of multipolar neurons displaying numerous spines along dendrites (SE *spiny neuron*s, 35.6% Fig. 3B, B′, C; EE *spiny* neurons, EE: 48.9%, Fig. 3B″, B′″–D. Additional file 1: Fig. S4; Additional file 1: Table S1). Interestingly, a fraction of spiny neurons (13.4%) displayed spine densities in the range of those reported for typical adult hMSNs (high spine density, *hsd,* neurons: more than 3.6 spines/10 μm dendrite; Graveland et al. [18]. Of note, in EE this proportion doubled (*p* = 0.042) and increased to about one-third (31.1%) of all inspected cells (Fig. 3C, D, Additional file 1: Fig. S4A, Additional file 1: Table S1). This finding suggests that EE promotes the acquisition of specific MSN traits. In addition, EE fostered a significant increment of spine numbers in transplanted *hsd* neurons compared to SE (SE 4.8 ± 0.3, EE 6.0 ± 0.4; *p* = 0.028; Fig. 3E–G), so that the mean spine density became closer to that reported for adult hMSNs (7.2 ± 1.2/10 μm, Graveland et al. [18]). These data support the selective action of EE on the promotion of a specific hMSN phenotype and suggest an increased integration of the grafted cells.

### Graft morphogenesis occurs through domain organization exhibiting striosomes-like features

**Fig. 4:**
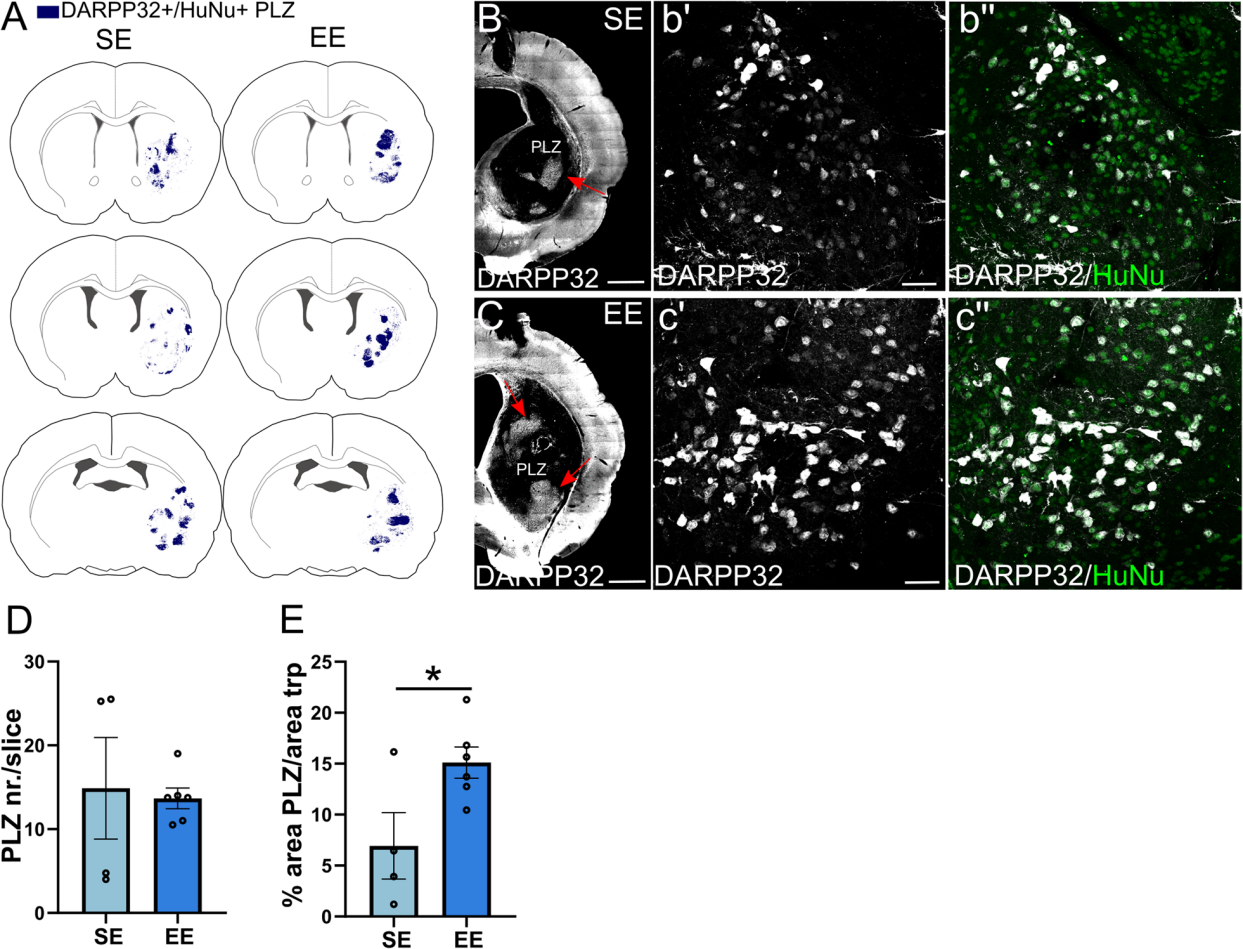
Analysis of human striatal graft compartmentalization. **A** Representative maps of the distribution of DARPP32^+^ cells in human striatal grafts. The fluorescent signal of DARPP32 staining was converted into a vector bitmap image by Inkscape software and overlapped with a schematic representation of rat brain coronal sections. **B** and **C** Representative images showing graft DARPP32^+^ neuron patch-like zones (PLZ; highlighted by the red arrowhead) in SE (**B**) and EE (**C**) housing. **b′** and **b″** High magnification confocal images illustrating clustered DARPP32-expressing HuNu^+^ neurons in SE grafts. **c′** and **c″** High magnification confocal images illustrating clustered DARPP32-expressing HuNu^+^ neurons in EE grafts. **D** Average numbers of DARPP32^+^ clusters per slice, in SE and EE conditions (unpaired *t* test, *p* > 0.05 ns; *N* = 4 SE, 6 EE). **E** DARPP32^+^ cluster size in EE compared to SE (unpaired *t* test, *p* < 0.05; *N* = 4 SE, 6 EE). Data are represented as mean ± SEM. Scale bars: 1 mm (**B**, **C**); 50 μm (**b′**–**c″**)

Here, we investigated the neurochemical organization of the developing human striatal grafts. At 6MPT, HuNu^+^/DARPP32^+^ cells appeared aggregated in clusters (patch-like zones, PLZ, Fig. 4A–C), which resembled the patch-zones corresponding to matured striatal tissue in primary fetal grafts obtained from either rat or human ganglionic eminences [26–28]. Similar clusters are known to emerge under CTIP2 control during striatal development in rodents [29]. This observation suggests that ESC-derived hMSNs self-organize and recapitulate key striatal developmental features. In human striatal grafts DARPP32^+^ PLZ were found in similar numbers (≃ 14 PLZ/slice) in EE compared to SE (Fig. 4D and Additional file 1: Table S1), yet they increased in size in EE, in agreement with more numerous DARPP32^+^ cells in the enriched grafts (*p* = 0.034; Fig. 4E and Additional file 1: Table S1). We speculated that regions outside PLZ could be enriched of cell types different from hMSNs and investigated the distribution of human CR^+^ interneurons, which are especially numerous in the primate striatum compared to rodents [30]. We found that CR^+^ interneurons also displayed a degree of segregation that did not overlap with DARPP32^+^ PLZ (Additional file 1: Fig. S5A). This pattern, which was not affected by the housing conditions (Additional file 1: Fig. S5A), suggests that at this stage of maturation human grafts are composed of domains with different developmental commitments.

Then, we asked if the human grafts displayed the neurochemically defined compartments of striosome and matrix. To detect the emergence of striosome-like features, we inspected the expression of the mu-Opioid receptor (muOR) [31, 32] (Additional file 1: Fig. S5B). In SE and EE grafts muOR positivity appeared to be similarly upregulated in groups of human cells distributed in PLZ (Additional file 1: Fig. S5C). Conversely, only a weak labeling was observed for the matrix marker Acetylcholine esterase (AChE; Brimblecombe et al. [31]) (Additional file 1: Fig. S5D–E).

Taken together, these observations suggest that human graft morphogenesis occurs through the formation of domains with distinct differentiation potencies and that striosome-like features emerge in the graft regardless of the housing conditions.

### Long-term transplanted human striatal progenitors innervate host striatal targets and receive both local and long-range input

We previously reported that grafted human striatal progenitor cells were able to extend fibers to innervate ipsilateral striatal target regions and as far as the substantia nigra (SN), showing a progressive increase in human projections from 1 to 2MPT along the antero-posterior axis [7]. At 6MPT we found that neurites from transplanted human cells, labeled with STEM121 and extending up to the ipsilateral SN, were maintained, with no differences between SE and EE in the percentage of SN covered by STEM121^+^ fibers (about 50%) (Additional file 1: Fig. S6A, B; Additional file 1: Table S1). In the SN, we observed human neurites reaching both pars reticulata (SNpr) and compacta (SNpc) (Additional file 1: Fig. S6a′–a″). Similar innervation in SE and EE was also found in the subthalamic nucleus (STN) (Additional file 1: Fig. S6C). Thus, long survival times and EE did not appear to significantly affect the distribution of graft projections as detected at 2MPT.

**Fig. 5:**
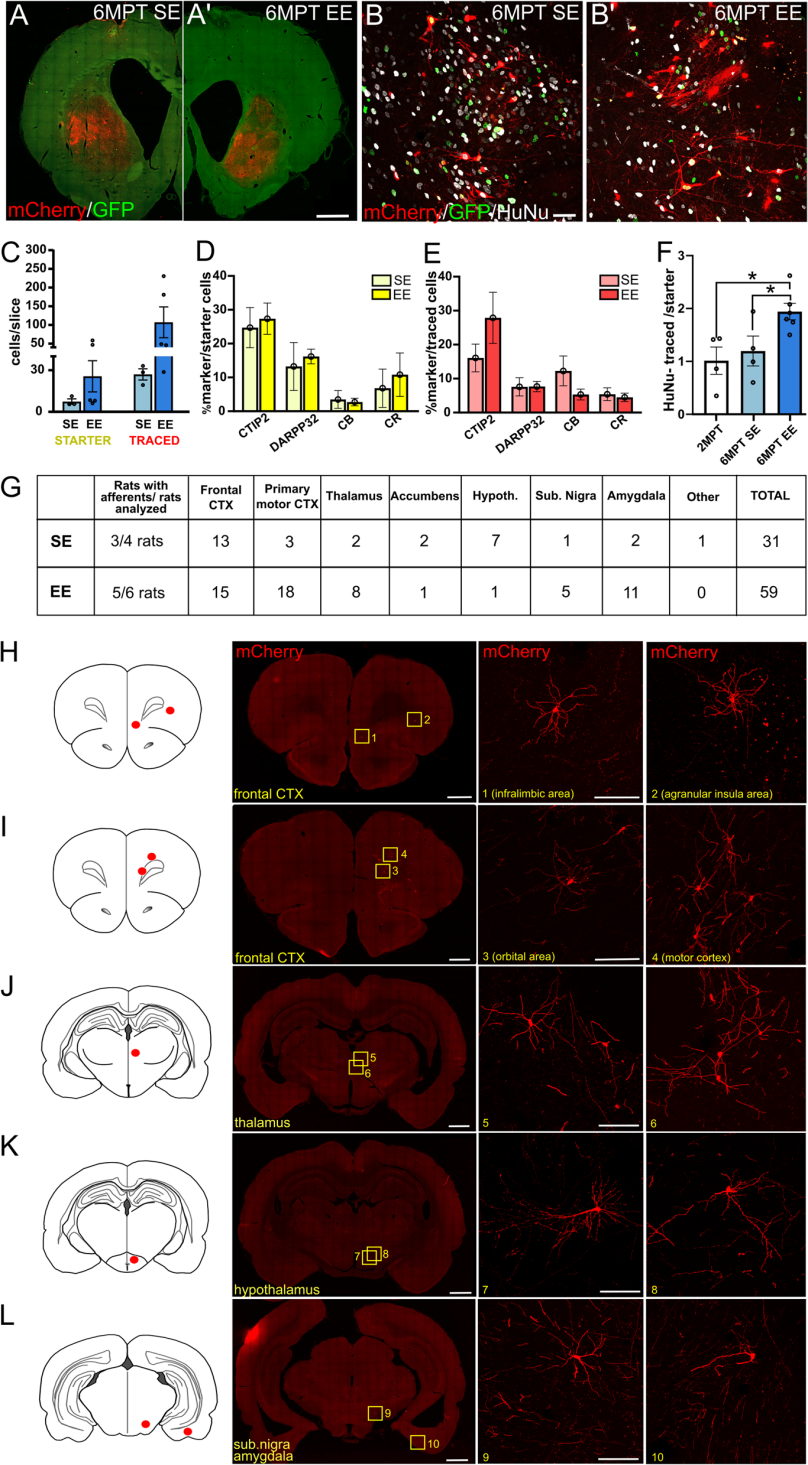
Analysis of local and long-range primary afferents to human striatal grafts at 6MPT. **A** and **A′** Representative images of both SE (left) and EE (right) striatal grafts in which mCherry-labeled cells are visible upon mRV transduction. **B** and **B′** High magnification confocal pictures of SE and EE transplants displaying viral-labeled mCherry-positive starter (GFP^+^) and traced (GFP^−^) cells. **C** Quantification of starter and traced cells per slice in SE and EE conditions (one way ANOVA, *p* > 0.05 ns. *N* = 4 SE; 6 EE). **D** and **E** Expression of striatal (CTIP2, DARPP32) or interneuron (CR, CB) markers in starter cells (**D**; two-way ANOVA, Group, *p* > 0.05 ns, markers, *p* < 0.01; *N* = 4 SE, 6 EE) and in mCherry^+^ input neurons (**E**; two-way ANOVA, Group, *p* > 0.05 ns, markers, *p* < 0.001; *N* = 4 SE, 6 EE), both from graft and host cells, in SE and EE conditions. **F** Quantification of host connections to starter neurons at 2 and 6MPT, in SE or EE housing (one way ANOVA, *p* < 0.05; Bonferroni post hoc test, 2MPT vs. 6MPT EE, *p* < 0.05; 6MPT SE vs. 6MPT EE, *p* < 0.05. *N* = 4 2MPT; 4 6MPT SE; 6 6MPT). **G** Number of grafted animals showing extrastriatal afferents and number of extrastriatal first afferents found in different brain regions in SE and EE conditions. **H**–**L** Schematic illustration of the antero-posterior distribution of extrastriatal host mCherry-positive first-order input neurons in SE and EE slices. Pictures display representative examples of extrastriatal rat neurons connecting to human grafted neurons and localized in diverse brain areas, such as the frontal cortex (**H**, **I**); thalamus (**J**), hypothalamus (**K**), substantia nigra and amygdala (**L**). Data are represented as mean ± SEM. Scale bars: 1 mm (**A** and tile-scans in **H**–**K**); 50 μm (**B**); 100 μm (high magnification in **H**–**K**)

To explore the establishment of host afferent inputs to the graft, we took advantage of a modified rabies virus-based tracing system (mRV) that allows selective tracing of first order afferents of transplanted human neurons (see “Methods” section; [7, 11]. By using this strategy, we found human starter cells in both SE and EE conditions (Fig. 5A–B′). Despite some variability, in the inspected sections starter cell densities were overall similar in the two housing conditions (Fig. 5C, Additional file 1: Table S1). Similarities in starter cell numbers in SE and EE were further confirmed by comparing the respective starter cell distribution densities via computed bootstrap confidence intervals of the mean with 100,000 runs, that resulted to be overlapping (SE, 3.04–10.4 vs. EE, 9.2–54.8). Starter cell fractions displaying MSN features such as CTIP2 or DARPP32 expression similarly dominated over other cell types (Fig. 5D; Additional file 1: Table S1).

We also observed numerous mCherry^+^ traced host neurons (mainly showing CTIP2-positivity; Fig. 5E; Additional file 1: Table S1) localized within the striatum, connecting to the grafted cells. Counting of the traced cells within the striatum in 6MPT animals exposed to EE resulted in 100.29 ± 40.7 traced cells/slice while only 21.44 ± 5.4 traced cells/slice were counted in SE (Fig. 5C). While these data reflect the positive increase of the Connectivity Index (computed as the percentage of traced over starter cells, SE, 3.04 ± 0.7; EE, 4.62 ± 0.4) in EE, however, these differences did not reach statistical significance (*p* = 0.06). Of note, the bootstrap confidence intervals of the mean on traced cell distribution turned out to be non-overlapping (SE, 25.5–40.25 vs. EE, 48.35–205.4), supporting the observed trend for a traced cell increase in EE. Moreover, when we distinguished traced cells between HuNu^+^ grafted versus HuNu^−^ host cells, we found that while the number of donor primary afferents did not vary between SE and EE (about 2 traced:1 starter cells in both conditions; Additional file 1: Table S1), host traced neurons (HuNu^−^/mCherry^+^ cells) at 6MPT increased significantly (1.5 fold) in EE compared to SE at both 6MPT and 2 MPT (*p* = 0.036; Fig. 5F and Additional file 1: Table S1), suggesting that EE favors the establishment of host-to-graft inputs.

Importantly, and contrary to findings reported at 2MPT [7], at 6MPT we found primary afferent host neurons in extra-striatal areas in 3 out of 4 inspected brains of the SE group, and in 5 out of 6 brains of the EE group (Table Fig. 5G), indicating the integration of human grafted neurons into long range host afferent circuits. Furthermore, primary afferent host neurons were distributed in ipsilateral areas known to project to the striatum such as frontal and motor cortices (infragranular layers), thalamus, hypothalamus and SN (Fig. 5H–L).

Connections from the cerebral cortex and thalamus were also confirmed by inspection of positivity for the vesicular glutamate transporters 1 (vGlut-1) or 2 (vGlut-2), respectively (Additional file 1: Fig. S7A, B). In line with data on extrastriatal primary afferents, results indicate a greater connectivity from the cortex than from the thalamus in both housing conditions.

TH positive fibers with beaded appearance likely belonging to host nigrostriatal afferents were also detected within the grafts where they formed dense meshwork especially close to the medial graft borders (Additional file 1: Fig. S7B–D′). These fibers appeared significantly increased in EE (*p* = 0.0056) (Additional file 1: Fig. S7B–D′ and Additional file 1: Table S1), indicating a supportive action of EE on the outgrowth of TH^+^ axons. However, the contribution of fibers of local TH^+^ interneurons cannot be excluded.

Taken together, these data show that human graft efferents to striatal targets are maintained up to 6 MPT and that at this late time point grafted striatal cells receive long-range inputs. They also suggest that EE further promotes the establishment of host-to-graft connections.

### Effect of long-term grafts and enriched environment on motor behavior

**Fig. 6:**
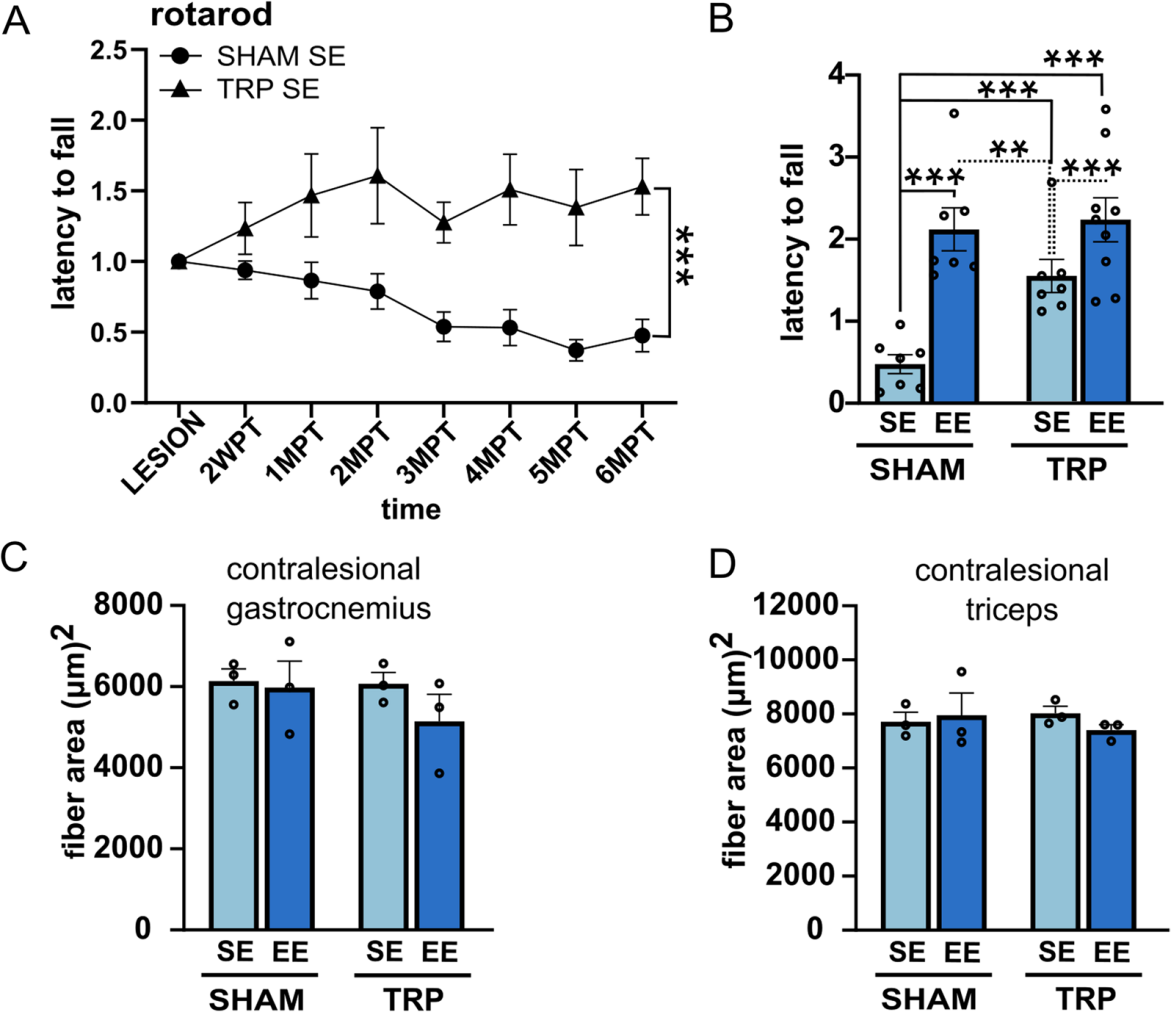
Long-term transplantation and enriched environment improve motor behavior with no effect on muscular trophism. **A** The graft alone improves complex motor performance as examined in the rotarod test compared to animals that were lesioned but did not receive the graft (SHAM) (two-way ANOVA, SHAM SE vs. TRP SE, Group: *F*_(1;96)_ = 58.48; *p* < 0.001). Data are normalized over ‘lesion’ values and represented as mean ± SEM. Asterisks indicate the main effects of the groups. See Additional file 1: Table S1 for post hoc analysis. **B** Exposure to EE improves motor recovery in SHAM rats, outperforms TRP SE condition and it equals the motor rescue obtained in EE grafted animals (two-way ANOVA, Group: *F*_(3;208)_ = 39.93; *p* < 0.001). 6MPT data normalized over “lesion” values are plotted. Data are represented as mean ± SEM. Asterisks indicate the main effects of the groups. See Additional file 1: Table S1 for additional information. *N* = 7 SHAM SE; 7 SHAM EE; 7 TRP SE; 9 TRP EE. **C** and **D** Measurements of muscle fiber area of contralesional gastrocnemius (**C**) and triceps (**D**) in the examined experimental groups and conditions (one way ANOVA, *p* > 0.05 ns); *N* = 3 SHAM SE; 3 SHAM EE; 3 TRP SE; 3 TRP EE. Data are represented as mean ± SEM

We had previously demonstrated that, already at 2MPT, human striatal grafts improve reflex-based sensorimotor responses associated with striatal circuits that were worsened by QA lesion [7]. In this study, we focused on the therapeutic potential of long-term engrafted human striatal progenitors, alone or in combination with EE, to rescue complex motor performances that did not show amelioration at 2MPT. After QA-induced lesion, latency to fall in the rotarod test was measured every month up to 6MPT in both sham and transplanted animals maintained in either SE or EE conditions. QA lesioned rats in SE displayed a progressive performance deterioration (Fig. 6A; SHAM SE: *y* = − 0.02568*x* + 0.9651; Slope *F*_(1;54)_ = 35.31, deviation from zero: *p* < 0.0001; Slope confidence intervals: − 0.03434 to − 0.01701). Human striatal graft significantly compensated such decline and the beneficial effect was measurable in all transplanted animals at all time points analyzed after 4MPT (TRP SE: *y* = 0.01252*x* + 1.241; Slope *F*(1;54) = 1.626, deviation from zero: *p* = 0.2078, Slope confidence intervals: − 0.007170 to 0.03222; two-way ANOVA, SHAM SE versus TRP SE, Group: *F*_(1;96)_ = 58.48; *p* < 0.001; see Additional file 1: Table S1 for post-hoc analysis) (Fig. 6A).

When we evaluated the impact of EE, we found that EE alone without graft improved task execution above the performance of TRP SE rats, with no further enhancement supported by the combination of the graft and EE at 6MPT (two-way ANOVA, Group: *F*_(3;208)_ = 39.93; *p* < 0.001; see Additional file 1: Table S1 for post hoc analysis) (Fig. 6B). These observations suggest that EE, in this behavioral test, despite enhancing morphological and phenotypic features of the grafted cells, does not further improve the graft therapeutic effect.

EE actions were not explained by increased muscle trophism, as, after examining skeletal muscle features, we found no changes in the cross-sectional area and Feret diameter of ipsi- (Additional file 1: Table S1) and contralesional skeletal muscle fibers between sham and transplanted animals, in both standard and enriched conditions (Fig. 6B, C).

Overall, these data show that over time transplantation of human striatal progenitors improves long-term complex motor performances impaired by QA-induced striatal degeneration. Also, EE improved task execution more than the graft alone, with no additive actions when combined with the graft.

### Tissue changes associated with EE

To look at possible factors accounting for increased maturation and integration of the human grafted neurons in EE, we focused on brain-derived neurotrophic factor (BDNF) and microglia. BDNF is a well-known player in activity-dependent neuronal plasticity and circuit remodeling that influences survival and differentiation of striatal MSNs [33, 34]. BDNF, whose transport and secretion are activity dependent [35], is mainly produced in the cortex and delivered to the striatum anterogradely via corticostriatal fibers [36].

**Fig. 7:**
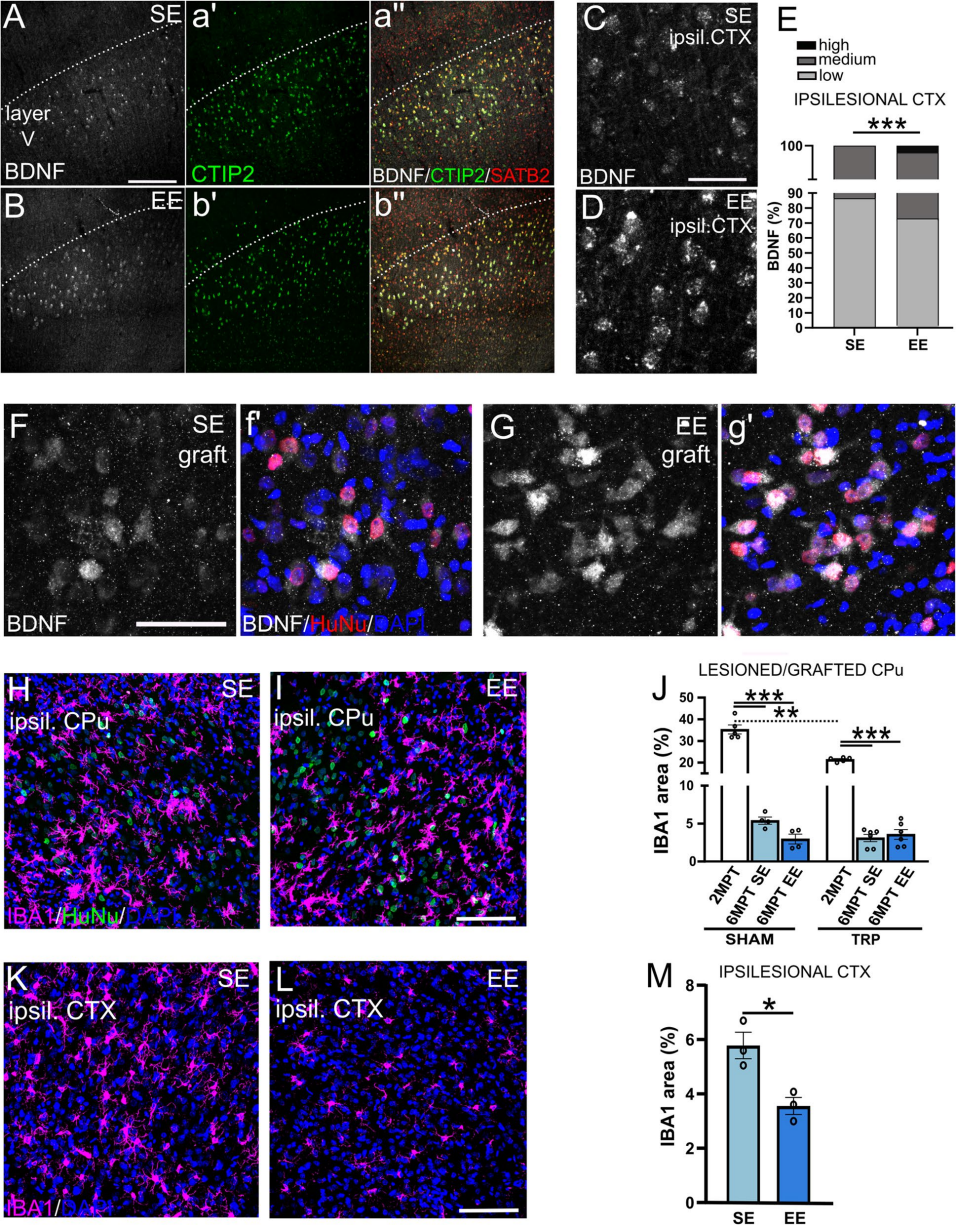
Analysis of BDNF and IBA1 expression levels in SE and EE housing conditions. **A** and **B** Expression of BDNF in the ipsilesional cortex of SE and EE housed animals, respectively. Cortical neurons are labeled with CTIP2 and SATB2 markers (**a'-b''**). **C** and **D** High-magnification images showing BDNF^+^ cortical neurons in SE and EE, respectively. **E** Proportions of cells with distinct BDNF staining intensities in SE and EE condition, in the ipsilesional cortex.  **F** and **G** High-magnification images showing BDNF expression in human (HuNu^+^) cells in the graft, in SE and EE conditions. **H** and **I** Representative images showing host IBA1-positive microglia (purple) in the grafted striatum of SE and EE animals. **J** Quantification of IBA1 expression in the striata (referring to the coordinates in the rat brain atlas) with and without grafts (SHAM) at short and long transplantation time and in SE and EE housing (one way ANOVA SHAM, *p* < 0.001. One way ANOVA TRP, *p* < 0.001. One-way ANOVA tot, *p* < 0.001. *N* = 5 SHAM 2MPT, 4 SHAM 6MPT SE; 4 SHAM 6MPT EE; 5 TRP 2MPT; 5 TRP 6MPT SE; 6 TRP 6MPT EE). **K** and **L** Representative images showing IBA1^+^ microglia in the ipsilesional cortex of SE and EE conditions. **M** The graph shows the quantification of IBA1 signal in the ipsilesional cortex (One-way ANOVA, *p* < 0.05. *N* = 3 rats SE, 3 EE). Data are represented as mean ± SEM. Scale bars: 200 μm (**A**); 50 μm (**C**; **F**); 100 μm (**I**; **L**)

Hence, we examined the intensity of BDNF protein levels in host cortical neurons of the ipsilesional grafted hemisphere and found a significant change in the distribution of BDNF weak, medium and strong intensity-expressing cells in EE versus SE (Fig. 7A–E). Specifically, in EE the frequency of cortical cells exhibiting medium or strong BDNF staining significantly increased compared to SE housing (Chi-square test *df*_(28;06;2)_, *p* < 0.001) (Fig. 7E; Additional file 1: Table S1).

BDNF appeared to increase also in grafted cells in EE compared to SE, which might suggest an incremented cortical supply to the graft after EE exposure (Fig. 7F, f′ and G, g′). Thus, EE appears to stimulate an upregulation of cortical BDNF, which, in turn, may support the maturation and integration of grafted hMSN precursors in EE.

In parallel, since we had found a relevant degree of microglia activation within the grafts at 2MPT [7], we examined microglia also at 6MPT, both within and outside the transplant, in comparison with QA-lesioned striata that did not receive the transplant (SHAM animals). Microglia IBA1-positive signal was significantly reduced at 6MPT compared to 2 MPT [7] in the striatum of both SHAM and transplanted groups in line with the progressive attenuation of tissue reactivity over time (Fig. 7J). However, within the graft microglia did not exhibit changes in morphology and IBA1^+^ area in SE versus EE (Fig. 7H–J, Additional file 1: Table S1). Outside the graft area, a significant reduction of about 40% in the area occupied by IBA1 signal was observed in the ipsilesional somatomotor cortex of EE-exposed animals compared to SE (Fig. 7K–M and Additional file 1: Table S1). These observations indicate that glial activation was progressively reduced over time and that EE modulates microglia in the host tissue.

## Discussion

This study characterizes a broad spectrum of morphological, molecular and functional properties related to long-term hESC-derived striatal grafts in a QA-based HD model. To the best of our knowledge, this manuscript is the first that examines the maturation, organization and connection reconstruction of hESC-derived striatal progenitors in a HD model and assesses the impact of environmental enrichment on human grafts.

We show that at 6MPT the grafts are composed of key striatal cell types including relevant fractions of matured MSNs, interneurons and astrocytes, which integrate into the host circuits by reaching proper targets and receiving long-range inputs, in addition to local ones. The grafts also show signs of compartmentalization and over-time ameliorate the execution of complex motor behaviors impaired by QA lesion. Furthermore, we prove that the acquisition of neurochemical and structural features of hMSNs and inputs from host neurons is further improved by exposure to EE housing.

After 6 months, the grafted cells displayed a negligible proliferation, ensuring the absence of uncontrolled overgrowth. This feature supports the safety of our cell replacement strategy and helps in removing a potential roadblock on the path to the clinic, which was highlighted by former studies [37]. Yet, at 6MPT a significant enlargement of the graft volume was detected in comparison with 2MPT. Growth of human cells and massive infiltration of neuropil by host astrocytes and microglia are likely major contributors to the detected graft size expansion, in line with data on clinical grade dopaminergic grafts [38].

After long-term transplantation, grafts repopulated the lesioned striatum and differentiated into several striatal cell types. A fraction of cells displayed clear MSN features, as qualified by the expression of a set of cell-type specific markers (CTIP2, DARPP32, GABA) and, most notably, by the acquisition of morphological traits distinctive of adult hMSNs [18]. The high density of dendritic spines also suggested a relevant degree of functional maturation in a fraction of cells. Further, peptides and dopamine receptors typical of direct or indirect pathway MSNs appeared expressed, with a broader presence of indirect pathway markers. This feature may constitute a therapeutic advantage in view of counterbalancing the initial preferential loss of indirect pathway MSNs and the hyperkinetic condition occurring in HD [39, 40]. However, the fraction of cells that displayed most mature MSN features remained limited. A higher yield of DARPP32^+^ MSNs has been previously reported [41], although in that case limitations apply due to marker expression inconsistencies in cell identity characterization. Yet, our scenario is consistent with the prolonged differentiation times of human cells compare to rodents, and the differentiation asynchrony may reflect the exposure to diverse local signals and/or extent and quality of the connectivity.

Grafts also included significant fractions of striatal interneurons. Such composition reminds of primary fetal striatal grafts derived from the entire ganglionic eminence [27, 42, 43]. The presence of interneurons may be important for a comprehensive reconstruction of the striatal circuitry because, in addition to MSNs, interneurons are also significantly affected in HD [44–46]. Also, it has been speculated that striatal interneurons could significantly contribute to support graft-mediated behavioral recovery [43, 47]. Further studies are needed to fully prove this possibility and assess whether and how the optimization of cell type graft composition can improve graft therapeutic efficacy.

Differentiation into the proper cell type(s) is an essential requisite for graft therapeutic actions. However, the complex structural and functional modularity of the striatum [27, 28, 31, 42, 48] suggests that graft functional impact may also be affected by the internal organization of the transplanted cells. While only partly recapitulating the mature striatal structure, fetal rat or human ganglionic eminence grafts display an arrangement in patch-zones comprising striatal neuron aggregates, and non-patch zones including non-striatal neuronal types [27, 43], with both aggregate types contributing to functional rescue. Our study shows that human grafts are also arranged according to a macroscopic modular architecture based on clustering of MSNs in patch-like zones, and the distribution of striatal interneurons in non-patch-like zones. Such pattern is suggestive of a compartmentalized graft development, where different compartments may reflect the specification of human progenitors to distinct ganglionic eminence territories [49, 50]. Such prevalent segregation may require longer times to be overcome and give rise to a fully mature tissue architecture. Further, the emergence of the striosome marker muOR within patch-like zones is in line with the earlier generation and maturation of striosome MSNs compared to those in the matrix [51–53]. Thus, grafted human striatal progenitors display self-organization properties and are engaged in physiologic morphogenetic processes.

Graft-dependent functional repair also requires the restoration of lost circuits by grafted neurons. We confirmed the capability of grafted striatal progenitors to both project to physiological striatal targets and receive relevant inputs. Long-term , grafts were much broadly connected with host circuits compared to short time post-grafting [7], as evidenced by the detection of first afferent neurons not only within the striatum but also in extrastriatal regions, with cortico-striatal projections representing the most frequent input. The full establishment of extrastriatal input synapses may require a degree of graft neuron maturation and/or favorable tissue conditions which are only achieved long-term (see below).

Former studies on rat primary ganglionic eminence fetal transplants showed that exposure to EE increased MSN features such as cell body size, spine density, and GABA positivity [10, 24, 54]. Our data demonstrate that also human striatal progenitors respond to EE complex stimulation and that EE selectively promotes the neurochemical and morphological maturation of grafted hMSNs, in parallel with increased host-to-graft connectivity. These findings further suggest that the grafted cells respond plastically to housing condition-dependent factors. Among mechanisms that underpin EE, neurotrophins expression is known to modify the host microenvironment by enhancing neuroplasticity [55, 56] and BDNF was proposed as a mediator of the impact of EE on rat fetal graft [54]. In accordance with this view, the observed increased cortical BDNF in EE may provide an additional BDNF supply to the graft via corticostriatal projections [36, 57], thus promoting cell maturation.

Microglia are additional players potentially affecting graft maturation and wiring. IBA1 signal declined in the grafted area over time suggesting a reduction in microglia hypertrophy and inflammatory state, which is in line with previous studies [55, 58, 59]. Given that prolonged microglia activation is considered maladaptive [60], attenuation of microglial activation in both the grafts and residual striatum could participate in favoring striatal re-innervation [61]. In addition, IBA1 signal downregulation in cortical areas of EE brains indicates a further action of EE to the host tissue, which in turn might also favor corticostriatal rewiring. EE is in fact known to induce a variety of morphological and functional changes in microglia which vary according to the context and to the brain region [62], but overall are interpreted as neuroprotective, anti-inflammatory, and supportive of circuit plasticity [55]. However, the specific microglia functional states in the graft and in the host tissue in SE and EE as well as their potential contribution to graft maturation and integration remain to be clarified.

Finally, while no significant improvement in the execution of complex motor programs was observed up to 2MPT [7], over time the graft supported these motor skills. This correlates with progressive maturation of the grafted hMSNs in conjunction with the establishment of long-range inputs onto the grafted cells—still missing at early time points [7]— from both the cortex and other extrastriatal areas. These features indicate a degree of rebuilding of the damaged circuits and could represent the substrate mediating the behavioral rescue, as observed in the transplanted animals in SE and EE conditions.

However, EE improved task execution to a higher degree than the graft alone, with no additive effect when combined with graft. Thus, EE, although showing a maturation and morphological effect on transplanted cells, does not appear to synergize with the therapeutic effect of the graft in this model system, at least in the functional recovery of this peculiar motor test. We can assume that EE would have the same positive effects on host endogenous striatal neurons that we have described for graft cells. Because host neurons outnumber graft cells, the motor defect might be mitigated in a graft independent manner as the graft contribution could be most likely too small to be detected compared to the effect driven by EE-enhanced host endogenous neurons. Also, EE could promote compensatory plasticity of parallel extrastriatal circuits. Yet, the precise demonstration of the contribution of graft connectivity and activity to behavioral amelioration awaits to be fully understood.

## Conclusions

To sum up, our study demonstrated that the transplantation of hESC-derived striatal progenitors supports functional motor recovery long-term by surviving, differentiating, self-organizing and connecting into the lesioned striatum. Our findings also demonstrate that non-invasive manipulations based on increased circuit activity are able to specifically accelerate hMSN differentiation and graft tissue integration. They also strongly support the authenticity and therapeutic value of human striatal grafts.

### Supplementary Information

**Additional file 1:** Additional Figures and Tables.

## Data Availability

The published article includes parts of the data used for specific analysis that are available in open access on the Zenodo platform (link: https://zenodo.org/communities/nscr/?page=1&size=20); the remaining data were generated or analyzed during this study and summarized in the accompanying tables, figures and additional materials. The data that support the findings of this study are available from the corresponding authors upon reasonable request. Further information and requests for resources and reagents should be directed to and will be fulfilled by the Lead Contact Annalisa Buffo (annalisa.buffo@unito.it). This study did not generate new unique reagents.

## Abbreviations

AChE: Acetylcholine esterase
BDNF: Brain-derived neurotrophic factor
CB: Calbindin
ChAT: Choline acetyltransferase
contral: Contralateral
Cpu: Striatum (caudato-putamen)
CR: Calretinin
CTX: Cortex
EE: Enriched environment
ENK: Enkephalin
GABA: Gamma aminobutyric acid
GFAP: Glial fibrillary acidic protein
GFP: Green fluorescent protein
H/E: Hematoxylin/eosin
HD: Huntington’s disease
hESCs: Human embryonic stem cells
hsd: High spine density neurons
HTT: Huntingtin gene
HuNu: Human nuclei
ipsil: Ipsilateral
LGE: Lateral ganglionic eminence
lsd: Low spine density neurons
MPT: Months post-transplantation
mRV: Mutated rabies virus
MSNs: Medium spiny neurons
muOR: Mu-opioid receptor
PFA: Paraformaldehyde
PLZ: Patches-like zones
PV: Parvalbumin
QA: Quinolinic acid
SE: Standard environment
SN: Substantia nigra
SNpc: Sub. nigra pars compacta
SNpr: Sub. nigra pars reticulata
STN: Subthalamic nucleus
SUBP: Substance P
TH: Tyrosine hydroxylase
TRP: Transplanted
v-Glut: Vesicular glutamate transporter
