# Supplementary material for: hESC-derived striatal progenitors grafted into a Huntington’s disease rat model support long-term functional motor recovery by differentiating, self-organizing and connecting into the lesioned striatum
Source: Stem Cell Res Ther. 2023 Jul 28;14:189. doi: 10.1186/s13287-023-03422-4 (PMC10386300; doi:10.1186/s13287-023-03422-4)
Supplement: Supplementary file 1 — Additional file 1: Additional Figures and Tables. [file 13287_2023_3422_MOESM1_ESM.pdf]

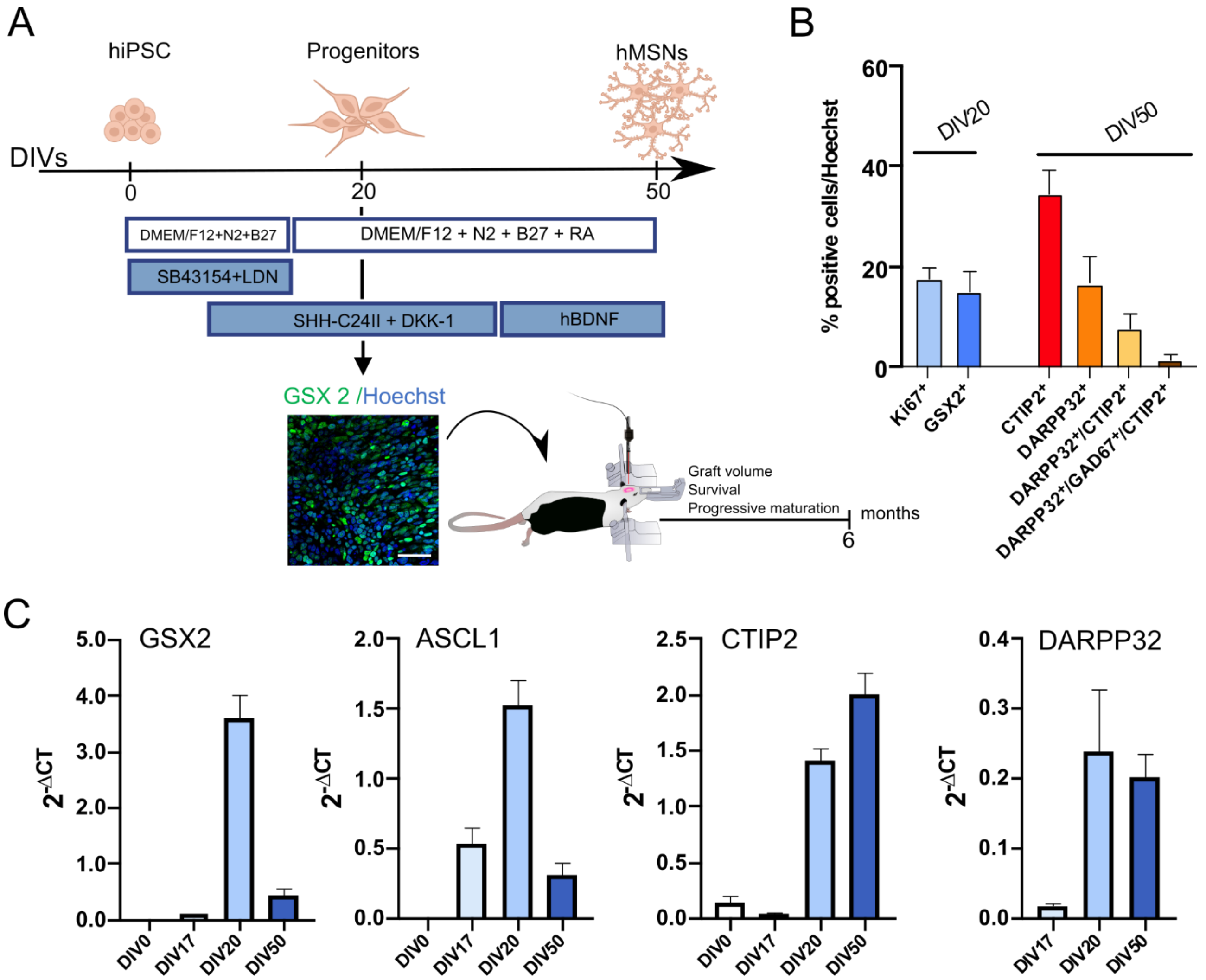

D

Gene	Forward (5' to 3')	Reverse (3' to 5')
GSX2	CTCGCTCATCATCAAGGACA	AGTGCAGGTGCGAAGTGA
ASCL1	GTCCTGTCGCCCACCATCTC	CCCTCCCAACGCCACTGAC
CTIP2	GAGTACTGCGGCAAGGTGTT	TAGTTGCACAGCTCGCACTT
DARPP32	CTGAGGACCAAGTGGAAGAC	GATGTCCCCTCCACTTCCTC
18S	CGGCTACCACATCCAAGGAA	GCTGGAATTACCGCGGCT

Additional Figure 1. In vitro differentiation procedures and quantification of hES cells differentiation (A) Schematic representation of the *in vitro* differentiation procedure (see material and methods for details) and representative image showing the expression of GSX2 at DIV20. (B) Quantification of the percentage of cells expressing Ki67, GSX2, CTIP2, DARPP32 and co-expressing DARPP32-CTIP2 or DARPP32-GAD67-CTIP2 over the total DAPI positive nuclei (N= 2 independent experiments, mean ±SEM). (C) Gene expression of selected striatal markers at different time points during the in vitro differentiation protocol (N= 2 independent experiments, mean ±SEM). (D) Table indicating the forward and reverse primers sequences used for the gene expression analyses of the selected striatal markers.

Scale bar: 50µm. Abbreviations: hiPSC, human pluripotent stem cells; hMSNs, human medium spiny neurons; DIV, days in vitro.

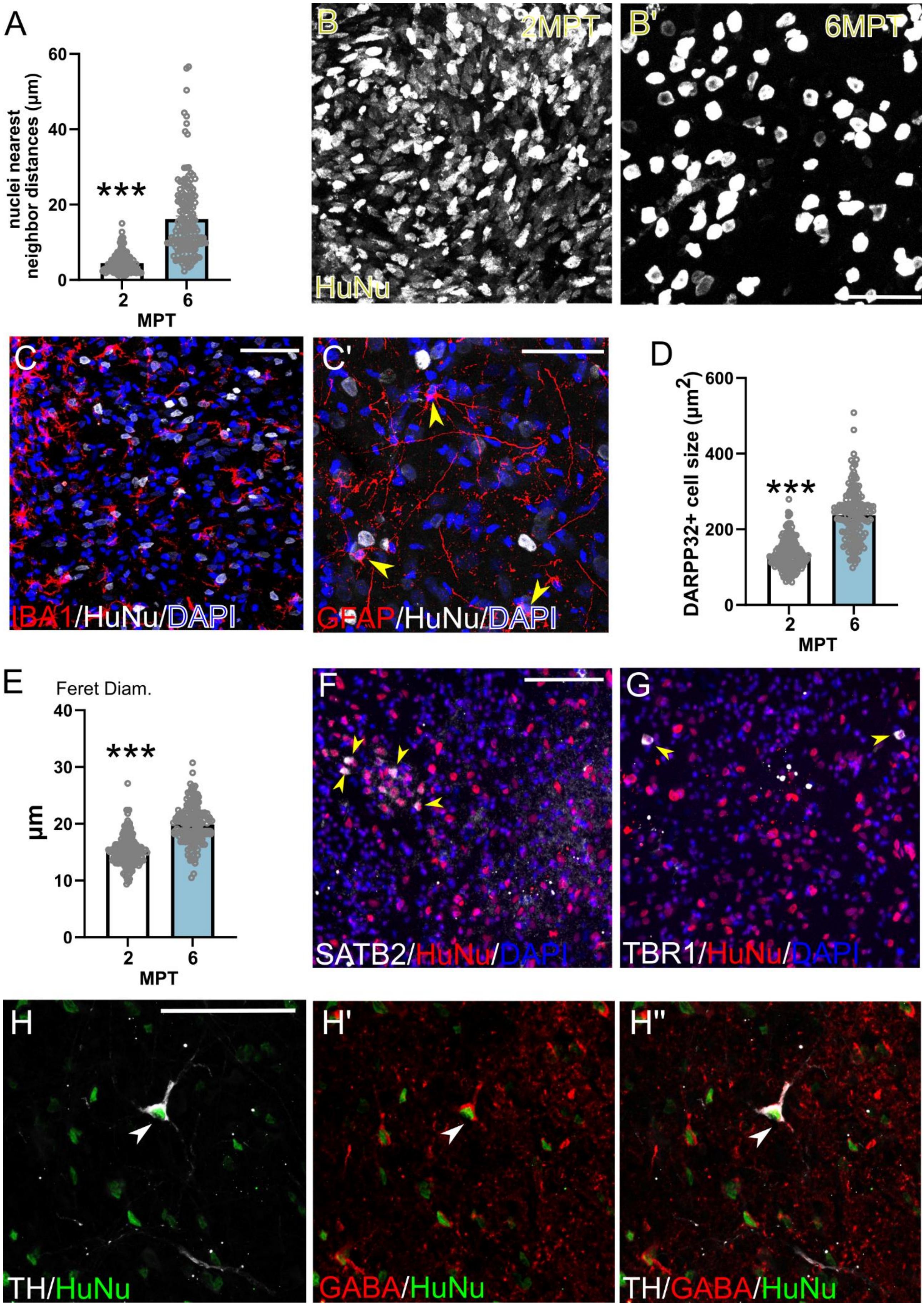
A
nuclei nearest neighbor distances (µm)
60
40
20
0
***
2
6
MPT
B
2MPT
HuNu
B'
6MPT
C
IBA1/HuNu/DAPI
C'
GFAP/HuNu/DAPI
D
DARPP32+ cell size (µm2)
600
400
200
0
***
2
6
MPT
E
Feret Diam.
µm
40
30
20
10
0
***
2
6
MPT
F
SATB2/HuNu/DAPI
G
TBR1/HuNu/DAPI
H
TH/HuNu
H'
GABA/HuNu
H''
TH/GABA/HuNu

Additional Figure 2. Comparison of short-term vs long-term human transplants. (A) Nearest neighbor distance of human nuclei shows increased distances at 6MPT compared to 2MPT (unpaired t-test, $p<0.001$; N=30 cells/animal; 6 animals 2MPT; 5 animals 6MPT SE). (B, B') Representative images showing differences in grafted cell distance between 2MPT (B) and 6MPT (B'). (C, C'') Representative images showing host IBA1^{+} microglial cells (C) and GFAP^{+} astrocytes (C') infiltrating the HuNu^{+} grafted cells. (D) Increased cell body area of hMSNs at 6MPT compared to 2MPT (unpaired t-test, $p<0.001$. N=30 cells/animal; 6 animals 2MPT; 5 animals 6MPT SE). (E) The feret diameter of hMSNs is also increased at 6MPT compared to 2MPT (unpaired t-test,$p<0.001$. N=30 cells/animal; 6 animals 2MPT; 5 animals 6MPT SE). (F, G) Representative image showing rare grafted cells expressing the cortical markers SATB2 (F, in white) or TBR1 (G, in white). (H, H', H'') Representative example of GABA positivity in a TH expressing grafted human interneuron. Data are represented as mean ± SEM. Scale bars: 50 µm (B', C, C'); 100 µm (F, H). Abbreviations: MPT, months post transplantation; HuNu, human nuclei; GFAP, glial fibrillary acidic protein; diam., diameter.

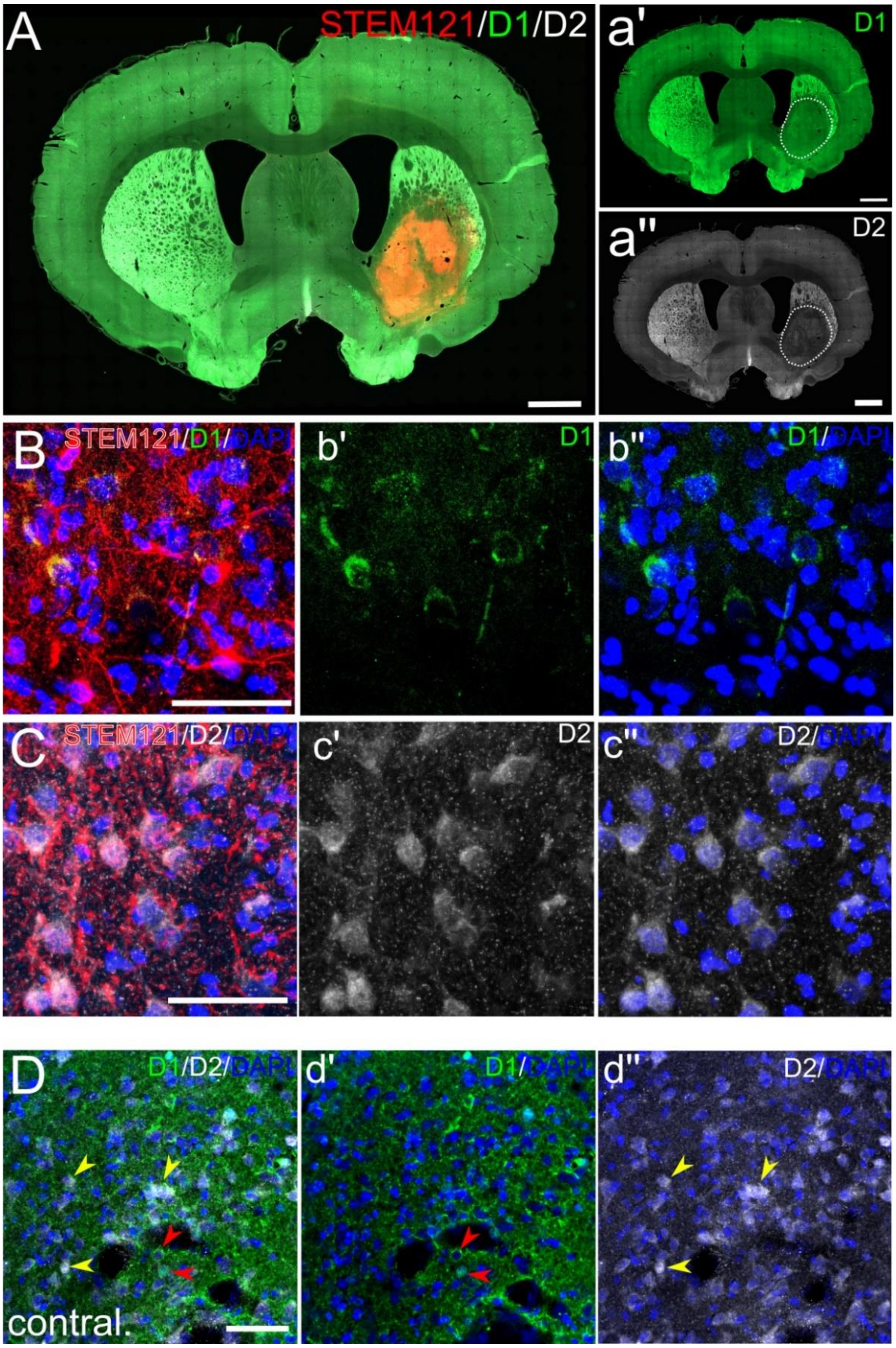

Additional Figure 3. hMSNs express neuropeptides commonly associated with striatal direct and indirect pathways. (A) The picture displays representative examples of the expression of human marker STEM121 (red) together with D1 (green) (a') or D2 (white) (a'') markers in the striatal region. (B) D1^{+} cells in STEM121^{+} grafted neurons. D1 expression is highlighted in panels (b'-b''). (C) D2^{+} cells in STEM121^{+} grafted neurons. D2 expression is highlighted in panels (c'-c''). (D) Picture displays the expression of D1 (d') and D2 (d'')

markers in the contralateral healthy striatal tissue. Arrowheads indicate some D1- (red arrowheads) or D2- (yellow arrowheads) positive cells. Scale bars: 1 mm (A, a', a'''); 50 µm (B, C, D). Abbreviations: D1, dopamine receptor type1; D2, dopamine receptor type 2; contral., contralateral.

A

	SE	EE
n (inspected neurons)	45	45
average soma size (µm^2 +/- SE)	178.6 +/- 12.2	205.3 +/- 12.2
Spiny neuron soma size (µm^2 +/- SE)	180.9 +/- 13.2	220.6 +/- 16.6
Aspiny neuron soma size (µm^2 +/- SE)	177.4 +/- 17.7	190.6 +/- 18.4
fractions of Spiny and high spine density neurons (%, n)	35.6; n=16<br>hsd neurons 13.4 n=6	48.8; n=22<br>hds neurons 31.1 n=14
fractions of Aspiny neurons (%, n)	64.4; n=29	51.1; n=23
primary dendrites (n +/- SE)	Average, 5.6 +/- 0.2<br>Spiny: from 3 to 9<br>Aspiny: from 3 to 9	Average, 5.9 +/- 0.2<br>Spiny: from 3 to 11<br>Aspiny: from 3 to 8
spine density per 10µm of high spine density neurons	4.0 +/- 0.4	6.1 +/- 0.2

B

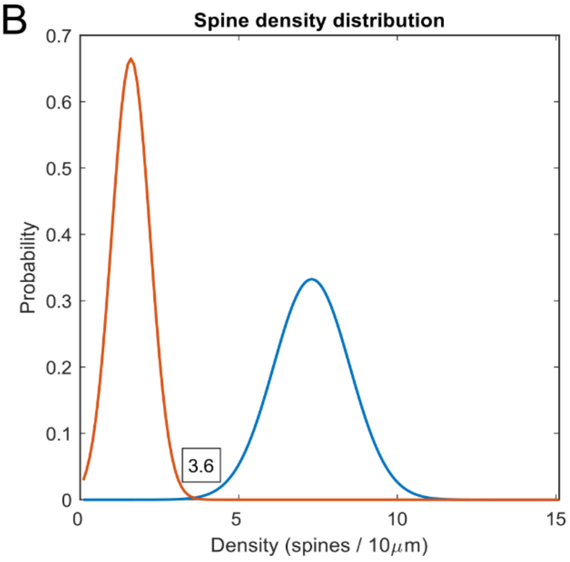

Additional Figure 4. Morphometric features of Spiny and Aspiny human grafted neurons in SE and EE rats. (A) Table reporting the morphometric features of graft Spiny and Aspiny cells in SE and EE conditions. (B) The curves illustrate the probability distribution function obtained from values of means and SD reported in Graveland et al., 1985. Abbreviations: SE, standard environment; EE, enriched environment; hsd; high spine density neurons.

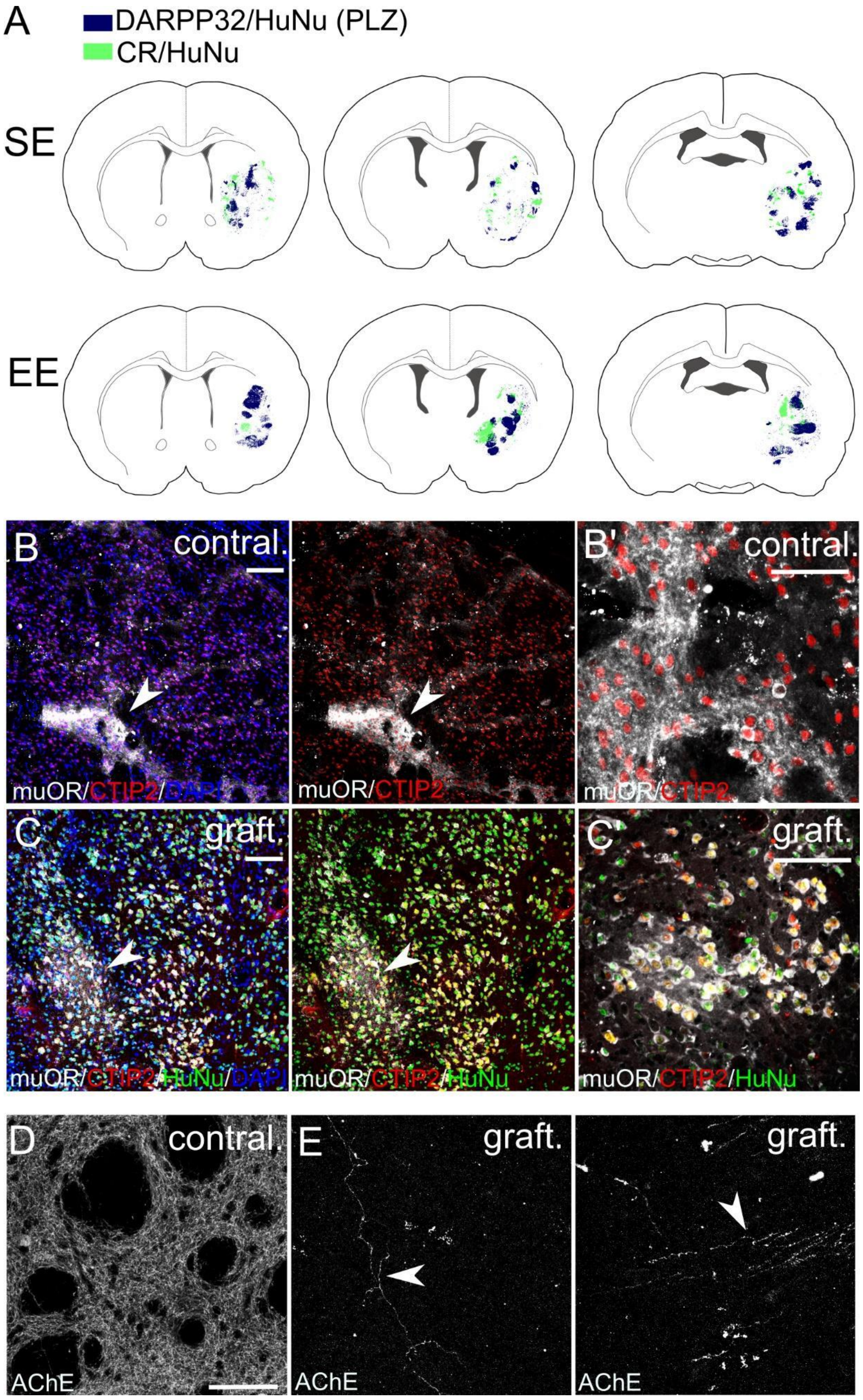
A
DARPP32/HuNu (PLZ)
CR/HuNu
SE
EE
B
contral.
muOR/CTIP2/DAPI
muOR/CTIP2
B'
contral.
muOR/CTIP2
C
graft.
muOR/CTIP2/HuNu/DAPI
muOR/CTIP2/HuNu
C'
graft.
muOR/CTIP2/HuNu
D
contral.
AChE
E
graft.
AChE
graft.
AChE

Additional Figure 5. Human grafts are organized in distinct domains and show muOR positivity. (A) Vector bitmap images (Inkscape software) mapping the fluorescent signal of DARPP32$^+$ and CR$^+$ cells in human striatal grafts. (B) Expression pattern of the mu-Opioid receptor (muOR) in the healthy contralateral rat striatum. White arrowhead indicates a striosome composed of muOR$^+$ cells. (B') High magnification image of a striosome structure in the contralateral striatum. Striatal CTIP2$^+$ cells (red) are expressing muOR. (C) Representative picture showing the expression pattern of muOR in SE grafts. White arrowhead highlights a striosome-like structure formed by human muOR+ cells. (C') Confocal high magnification picture of a striosome-like structure formed by grafted HuNu$^+$ cells (green) co-expressing CTIP2 (red) and muOR (white). No relevant differences were found in muOR expression pattern in EE compared to SE (not shown). (D) Confocal image showing the expression of Acetylcholine esterase (AChE) in the healthy contralateral rat striatum. (E) Few AChE$^+$ fibers are observed in the grafted striata. Scale bars: 100 μm (B, C); 50 μm (D). Abbreviations: HuNu, human nuclei; CR, calretinin; muOR, mu-Opioid receptor; AChE, Acetylcholine esterase; contral., contralateral; graft., grafted.

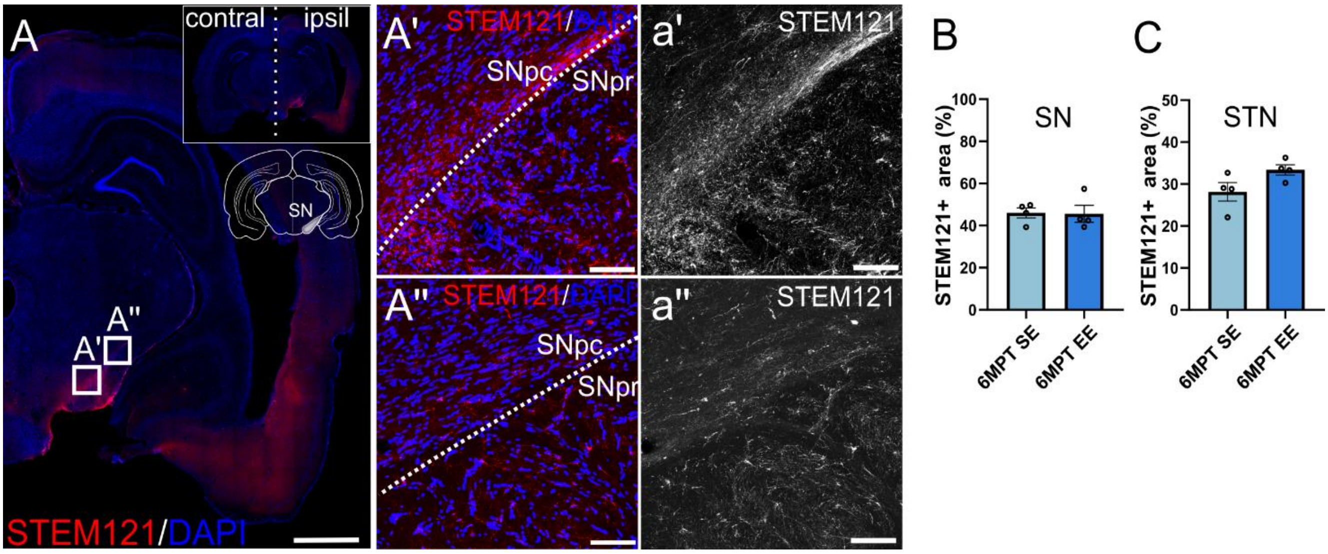

Additional Figure 6. Grafted hMSNs innervate striatal target regions up to 6MPT. (A) STEM121$^+$ human neurites (in red) extend up to the ipsilateral substantia nigra (SN), including both pars compacta and reticulata in an animal maintained in SE. Insets (A' and A'') show STEM121 fibers (red signals) in the SN. The same fibers are highlighted in white in (a'-a''). (B) Percentage of SN area covered by STEM121$^+$ fibers in SE or EE conditions (unpaired t-test, $p>0.05$ ns. N=4 SE, 4 EE). (C) No differences are found in the percentage of STN area covered by STEM121$^+$ fibers in SE or EE conditions (unpaired t-test, $p>0.05$ ns. N=4 SE, 4 EE). Scale bars: 1mm (A); 100μm (A'-a'; A''-a''). Data are represented as mean ± SEM. Abbreviations: contral., contralateral; ipsil, ipsilateral; SN, substantia nigra;

SNpc, substantia nigra pars compacta; SNpr, substantia nigra pars reticulata; STN, subthalamic nucleus; SE, standard environment, EE; enriched environment.

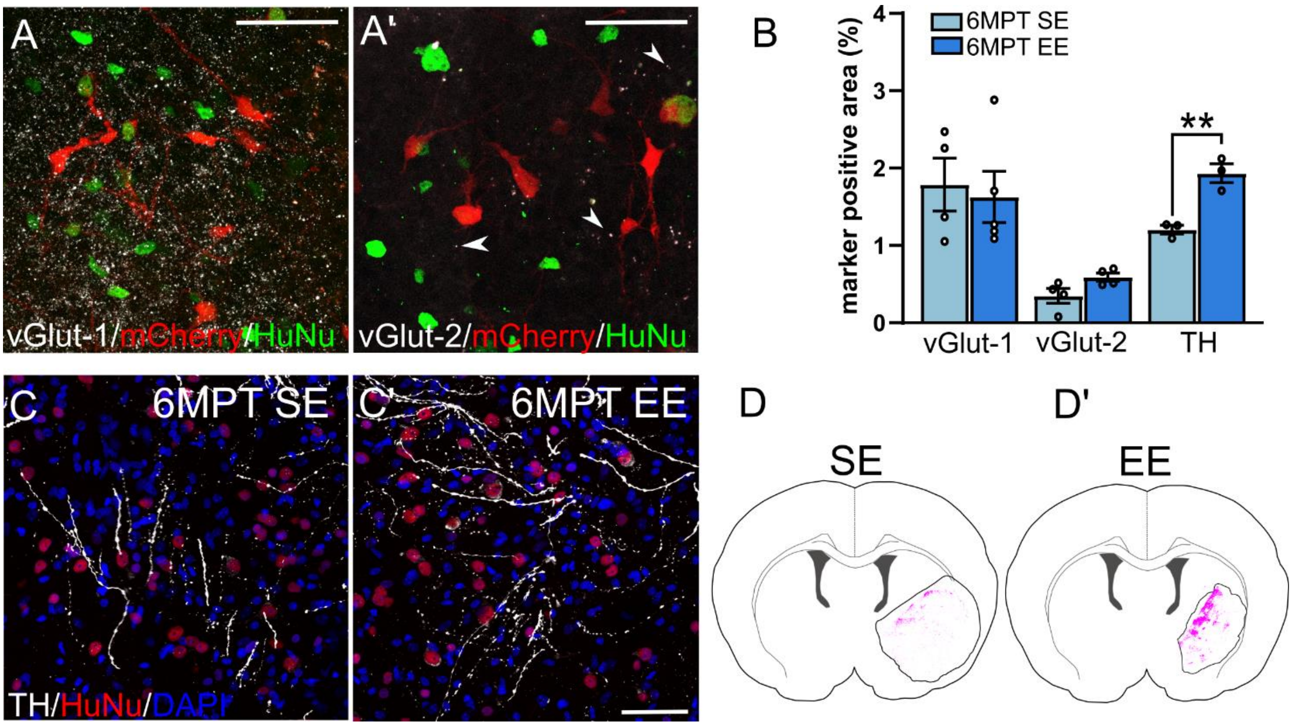

Additional Figure 7. Terminals of cortical and thalamic afferents and dopaminergic innervation in the graft at 6MPT. (A-A') Innervation from cerebral cortex or thalamus is confirmed by the presence of the vesicular glutamate transporter 1 (vGlut-1) or 2 (vGlut-2), respectively, in the graft region (white dots). White arrowheads in (A') indicate vGlut-2$^+$ dots in the graft region. (B) Percentage of area immunoreactive for vGlut-1, vGlut-2 and Tyrosine Hydroxylase (TH) in the graft (vGlut-1 and 2, unpaired t-test, $p>0.05$ ns. N= 4 SE, 5 EE. TH, Wilcoxon-Mann-Whitney Test, $p<0.01$. N=3 SE, 3 EE). (C-C') Increased density of TH$^+$ fibers in the graft in EE housing condition compared to SE. (D-D') Representative vector bitmap images (Inkscape software) mapping the fluorescent signal for TH in the grafted striatum. Data are represented as mean ± SEM. Scale bars: 50 µm. Abbreviations: vGlut-, vesicular glutamate transporter; TH, Tyrosine Hydroxylase; SE, standard environment; EE, enriched environment.

Figure	Experimental datasets	Statistical details and n of samples
Figures 1, 2, Additional 2	Graft size, cell density and dimensions.	**Figures 1A and 2A** Graft size: 2MT, 6.53 ± 0.6 mm^3; 6MPT SE, 40.7 ± 14.6 mm^3; 6MPT EE 32.7 ± 7.04 mm^3. One way ANOVA $F_{(2;14)}$=4.218; Bonferroni post hoc test, 2MPT vs 6MPT SE, p<0.001; 2MPT vs 6MPT EE, p<0.01.Unpaired t-test, 2MPT vs 6MPT SE, p=0.03; unpaired t-test 6MPT SE vs 6 MPT EE, p>0.05 ns. N= 6 2MPT; 5 6MPT SE, 6 6MPT EE. Estimated cell fraction over grafted cells: 2MPT, 78.6 ± 11.2%; 6MPT SE, 76.88 ± 27.9%; 6MPT EE, 68.9 ± 19.3%. One way ANOVA, $F_{(2;13)}$= 0.068, p=0.9344 ns. Unpaired t-test, 2MPT vs 6MPT SE, p>0.05 ns; unpaired t-test 6MPT SE vs 6 MPT EE, p>0.05 ns. N= 6 2MPT; 5 6MPT SE; 6 6MPT EE.<br>**Figures 1B and 2B** Cell area density: 2MPT, 8905.2 ± 643.9 cells/mm2, 6MPT SE, 1366.5 ± 114.4 cells/mm^2; 6MPT EE, 1439.8 ± 128.1 cells/mm^2. One way ANOVA, $F_{(2;13)}$=104.9. Bonferroni post hoc test, 2MPT vs 6MPT SE, p<0.001; 2MPT vs 6MPT EE, p<0.001. Unpaired t-test, 2MPT vs 6MPT SE, p<0.001; unpaired t-test 6MPT SE vs 6 MPT EE, p>0.05 ns. N= 6 2MPT; 4 6MPT SE; 6 6MPT EE.<br>**Figures 2C and Additional 2A** Nearest neighbor distances: 2MPT, 4.47µ ± 0.2; 6MPT SE, 16.17µ ± 0.8; 6MPT EE, 18.42µ ± 0.8. One way ANOVA, $F_{(2;537)}$=125.4, p<0.001. Bonferroni post hoc test, 2MPT vs 6MPT SE, p<0.001; 2MPT vs 6MPT EE, p<0.001. Unpaired t-test, 2MPT vs 6MPT SE, p<0.001; unpaired t-test 6MPT SE vs 6 MPT EE, p>0.05 ns. N=30 cells/animal; 2MPT, 6 animals; 6MPT SE, 5 animals; 6MPT EE, 6 animals.<br>**Figures 2K, Additional 2D, Additional 2E** DARPP32+ cell body area: 2MPT: 133.80$µ^2$ ± 2.9; 6MPT SE: 237.13$µ^2$ ± 5.7; MPT EE: 279.06$µ^2$ ± 5.6; feret diam., 2MPT: 15.23µ ± 0.2; 6MPT SE: 19.88µ ± 0.3; 6MPT EE: 21.51µ ± 0.2. One way ANOVA, $F_{(2;537)}$=240.0, p<0.001. Bonferroni post hoc test, 2M PT vs 6MPT SE, p<0.001; 2MPT vs 6MPT EE, p<0.001; 6MPT SE vs 6MPT EE, p<0.001. Unpaired t-test, 2MPT vs 6MPT SE, p<0.001; unpaired t-test 6MPT SE vs 6 MPT EE, p<0.001. N=30 cells/animal; 2MPT, 6 animals; 6MPT SE, 5 animals; 6MPT EE, 7 animals.
Figures 1, 2, Additional 3	Marker expression in grafted cell (on $HuNu^+$ population)	**Figures 1D and 2D** ki67: 2MPT, 8.69 ± 1.1%; 6MPT SE, 0.31 ± 0.08%; 6MPT EE, 0.65 ± 0.18%. One way ANOVA, $F_{(2, 11)}$=33.38; Bonferroni post hoc test, 2MPT vs 6MPT SE, p<0.001; 2MPT vs 6MPT EE, p=0.0001. Unpaired t-test, 2MPT vs 6MPT SE, p<0.01; unpaired t-test 6MPT SE vs 6 MPT EE, p>0.05 ns. N=6 2MPT, 4 6MPT SE, 4 6MPT EE.<br>**Figures 1D and 2D** NESTIN: 2MPT, 67.80 ± 4.5%; 6MPT SE, 16.07 ± 2.1%; 6MPT EE, 14.97 ± 1.03%; One way ANOVA, $F_{(2, 11)}$=76.99; Bonferroni post hoc test, 2MPT vs 6MPT SE, p<0.001; 2MPT vs 6MPT EE, p=0.0001. Unpaired t-test, 2MPT vs 6MPT SE, p<0.001; unpaired t-test 6MPT SE vs 6 MPT EE, p>0.05 ns. N=6 2MPT, 4 6MPT SE, 4 6MPT EE.<br>**Figures 1E and 2H** CTIP2: 2MPT, 45.3 ± 1.94%; 6MPT SE, 51.84 ± 7.35%; 6MPT EE, 55.54 ± 3.4%; One way ANOVA, $F_{(2, 15)}$=1.526; Bonferroni post hoc tests p>0.05 ns. Unpaired t-test, 2MPT vs 6MPT SE, p>0.05 ns; unpaired t-test 6MPT

		SE vs 6 MPT EE, p>0.05 ns. N=6 2MPT, 5 6MPT SE, 7 6MPT EE.<br>**Figures 1E and 2H** CTIP2/DARPP32: 2MPT, 2.8 ± 0.3%; 6MPT SE, 10.99 ± 1.3%; 6MPT EE, 20.3 ± 1.25%; One way ANOVA, $F_{(2, 15)}$=71.40; Bonferroni post hoc test, 2MPT vs 6MPT SE, p>0.05 ns; 2MPT vs 6MPT EE, p<0.001; 6MPT SE vs 6MPT EE, p<0.001. Unpaired t-test, 2MPT vs 6MPT SE, p<0.001; unpaired t-test 6MPT SE vs 6 MPT EE, p=0.027. N=6 2MPT, 5 6MPT SE, 7 6MPT EE.<br>**Figures 1E and 2H** GABA: 2MPT, 28.19 ± 0.8%; 6MPT SE, 32.38 ± 3.8%; 6MPT EE, 49.77 ± 4.3%; One way ANOVA, $F_{(2, 12)}$=14.54; Bonferroni post hoc test, 2MPT vs 6MPT SE, p=0.0004; 2MPT vs 6MPT EE, p=0.0007; 6MPT SE vs 6MPT EE, p=0.0078. Unpaired t-test, 2MPT vs 6MPT SE, p>0.05 ns; unpaired t-test 6MPT SE vs 6 MPT EE, p=0.023. N=6 2MPT, 4 6MPT SE, 5 6MPT EE.<br>**Figures 1G and 2E** CB: 2MPT, 1.83 ± 0.1%; 6MPT SE, 9.78 ± 1.9%; 6MPT EE, 10.69 ± 1.3%; One way ANOVA, $F_{(2, 11)}$=20.62; Bonferroni post hoc test, 2MPT vs 6MPT SE, p<0.01; 2MPT vs 6MPT EE, p<0.001. Unpaired t-test, 2MPT vs 6MPT SE, p<0.001; unpaired t-test 6MPT SE vs 6 MPT EE, p>0.05 ns. N=6 2MPT, 4 6MPT SE, 4 6MPT EE.<br>**Figures 1G and 2E** CR: 2MPT, 2.61 ± 0.4%; 6MPT SE, 13.55 ± 2%; 6MPT EE, 11.18 ± 0.7%; One way ANOVA, $F_{(2, 11)}$=26.04; Bonferroni post hoc test, 2MPT vs 6MPT SE, p<0.001; 2MPT vs 6MPT EE, p<0.001. Unpaired t-test, 2MPT vs 6MPT SE, p<0.001; unpaired t-test 6MPT SE vs 6 MPT EE, p>0.05 ns. N=6 2MPT, 4 6MPT SE, 4 6MPT EE.<br>**Figures 1G and 2E** TH: 2MPT, 0.91 ± 0.2%; 6MPT SE, 18.53 ± 2.6%; 6MPT EE, 9.65 ± 3.1%; One way ANOVA, $F_{(2, 7)}$=18.42; Bonferroni post hoc test, 2MPT vs 6MPT SE, p<0.01; 2MPT vs 6MPT EE, p<0.01. Unpaired t-test, 2MPT vs 6MPT SE, p<0.001; unpaired t-test 6MPT SE vs 6 MPT EE, p>0.05 ns. N= 4 2MPT, 3 6MPT SE, 3 6MPT EE.<br>**Figures 1D and 2D** GFAP: 2MPT, 2.14 ± 0.2%; 6MPT SE, 5.21 ± 1.2%; 6MPT EE, 3.96 ± 1.5%; One way ANOVA, $F_{(2, 9)}$=3.906; p>0.05 ns. Unpaired t-test, 2MPT vs 6MPT SE, p=0.0091; unpaired t-test 6MPT SE vs 6 MPT EE, p>0.05 ns. N= 6 2MPT, 3 6MPT SE, 3 6MPT EE.<br>**Figures 1G and 2E** PV: 6MPT SE, 3.16± 1.3%; 6MPT EE, 1.67 ± 0.6%; unpaired t-test, p=0.3618 ns. N= 3 6MPT SE, 3 6MPT EE.<br>**Figure 2E** ChAT: 6MPT SE, 11.74± 1.1%; 6MPT EE, 12.24 ± 0.9%; unpaired t-test, p=0.7425 ns. N= 3 6MPT SE, 3 6MPT EE.<br>**Figure 2J** SUBP and ENK: SUBP, 6MPT SE, 7.96 ± 2.1%; 6MPT EE, 6.45 ± 0.9%. ENK, 6MPT SE, 22.09 ± 0.3%; 6MPT EE, 24.37 ± 2.9%. One way ANOVA, $F_{(3,8)}$=25.05, p=0.0002. Bonferroni post hoc test, 6MPT SE ENK vs 6MPT SE SUBP, p=0.0030; 6MPT EE ENK vs 6MPT EE SUBP, p=0.0006. N=3 6MPT SE, 3 6MPT EE.
Figure 3	Maturation of hMSNs	**Figures 3C, 3D** Morphology: Aspiny, 6 MPT SE, 64.4% (N= 29 cells), 6 MPT EE, 51.1% (N= 23 cells). Spiny, 6MPT SE:35.6% (N= 16 cells), 6MPT EE:48.9% (N= 22 cells). Aspiny vs Spiny SE vs EE, Chi-square test, p=0.230. *hsd neurons*, 6 MPT SE, 13.4% (N= 6 cells), 6 MPT EE, 31.1% (N= 14 cells). Chi-square test, *hsd neurons* vs others SE vs EE, p= 0.042. N= 45 cells SE; 45 cells EE.

		**Figure 3G** Spine number: SE 4.8 ± 0.3, EE 6.0 ± 0.4; one-tailed Mann-Whitney test, p= 0.028. SE N= 54 dendrites, 14 cells (4 rats). EE, N=68 dendrites, 15 cells (4 rats).
Figure 4	Developmental morphogenesis of the graft	**Figure 4D** DARPP32+ patch-like zones (PLZ) number: SE, 14.87 ± 6.1 clusters; EE: 13.67 ± 1.2 clusters; Unpaired t-test, p>0.05. N=4 SE; 6 EE.<br>**Figure 4E** DARPP32+ patch-like zones (PLZ) size: SE, 6.93% ± 3.2 DARPP32+ area/graft area; EE, 15.11% ± 1.5 DARPP32+ area/graft area; Unpaired t-test, p=0.034. N=4 SE; 6 EE.
Figures 5, Additional 6, Additional 7	Local and long-range input to the graft	**Figures Additional 6B and 6C** Host to graft connections: SN STEM121+ area, SE: 47.72 ± 3.07%; EE: 46.95 ± 5.4%; Unpaired t-test, p>0.05 ns. STN STEM121+ area, SE: 28.15 ± 2.2%; EE: 33.38 ± 1.2%; unpaired t-test, p>0.05 ns. N=4 SE, 4 EE.<br>**Figure 5C** Starter and traced per slice: 6MPT SE: 6.75 ± 2.02 starter/slice; 21.44 ± 5.4 traced/slice; 6MPT EE: 25.15 ± 11.5 starter/slice; 100.29 ± 40.7 traced/slice. One way ANOVA, $F_{(3;16)}$=2.834; p=0.0713 ns. N= 4 SE; 6 EE.<br>Starter distribution densities via computed bootstrap confidence intervals of the mean (100000 runs): SE, 3.04-10.4; EE, 9.2-54.8. Traced distribution densities via computed bootstrap confidence intervals of the mean (100000 runs): SE, 25.5-40.25: EE, 48.35-205.4.<br>**Figure 5D** Marker expression in starter cells: CTIP2, SE: 24.72 ± 5.9%; EE: 27.34 ± 4.6%. DARPP32, SE: 13.24 ± 7.1%; EE: 16.19 ± 2.2%; CB, SE: 2.63 ± 2.7%; EE: 2.70 ± 1.1%; CR, SE: 6.81 ± 5.6%; EE: 10.80 ± 6.4%. Two way ANOVA, Group, $F_{(1;32)}$=0.4176; p=0.5227 ns; Markers, $F_{(3;32)}$=8.325; p=0.003. Sidak's multiple comparisons test, CTIP2 vs CB, p=0.0002; CTIP2 vs CR, p=0.0066; other comparisons ns. N= 4 SE, 6 EE.<br>Connectivity index: SE, 3.04 ± 0.7; EE, 4.62 ± 0.4, Unpaired t-test, p=0.06 ns.<br>**Figure 5E** Marker expression in traced cells: CTIP2, SE: 16.08 ± 4.08%; EE: 27.89 ± 7.5%. DARPP32, SE: 7.594 ± 2.7%; EE: 7.67 ± 1.5%; CB, SE: 12.26 ± 4.4%; EE: 5.32 ± 1.6%; CR, SE: 5.39 ± 1.9%; EE: 4.48 ± 1.2%. Two way ANOVA, Group, $F_{(1;32)}$=0.1277; p=0.723 ns; Markers, $F_{(3;32)}$=7.249; p=0.0008. N= 4 SE, 6 EE.<br>Human primary afferents/slice: 2MPT, 5.64 ± 1.02; 6MPT SE, 1.84 ±0.40; 6MPT EE, 2.51 ± 0.26. One way ANOVA, $F_{(2;11)}$=11.31, p=0.0021. Bonferroni post hoc test, 2MPT vs 6MPT SE, p=0.0033; 2MPT vs 6MPT EE, p=0.0069; 6MPT SE vs 6MPT EE, p>0.05 ns. N= 4 2MPT; 4 6MPT SE; 6 6MPT EE.<br>**Figure 5F** Host primary afferents/slice: 2MPT, 1.01 ± 0.26; 6MPT SE, 1.19 ±0.3; 6MPT EE, 1.94 ± 0.15. One way ANOVA, $F_{(2;11)}$=5.447, p=0.0227. Bonferroni post hoc test, 2MPT vs 6MPT SE, p>0.05 ns; 2MPT vs 6MPT EE, p=0.036; 6MPT SE vs 6MPT EE, p=0.0362. N= 4 2MPT; 4 6MPT SE; 6 6MPT EE.<br>**Figure S7B** Afferent connection markers: vGlut-1 signal intensity, 6MPT SE, 1.79 ± 0.34%; 6MPT EE, 1.63 ± 0.3%. Unpaired t-test, p>0.05 ns. N= 4 SE, 4 EE. vGlut-2 signal intensity, 6MPT SE, 0.35 ± 0.09%; 6MPT EE, 0.59 ± 0.05%. Unpaired t-test, p>0.05 ns. N=4 SE, 5 EE. TH signal intensity:

		6MPT SE, 1.2 ± 0.06%; 6MPT EE, 1.93 ± 0.1%. Wilcoxon-Mann-Whitney Test, p=0.0056. N=3 SE, 3 EE.
Figure 6	Behavior and related analysis	**Figure 6A, 6B** Rotarod:<br>Latency to fall, mean ± s.e.m: (2WPT), SHAM SE: 0.94 ±0.07; TRP SE: 1.23 ±0.18; SHAM EE: 1.56± 0.18; TRP EE: 1.74 ±0.33. (1MPT), SHAM SE: 0.87 ±0.13; TRP SE: 1.47 ±0.29; SHAM EE: 1.42± 0.16; TRP EE: 1.50 ±0.23. (2MPT), SHAM SE: 0.79 ±0.13; TRP SE: 1.61 ±0.34; SHAM EE: 1.93± 0.44; TRP EE: 2.21 ±0.38. (3MPT), SHAM SE: 0.54 ±0.10; TRP SE: 1.27 ±0.14; SHAM EE: 1.83± 0.27; TRP EE: 2.15 ±0.33. (4MPT), SHAM SE: 0.53 ±0.13; TRP SE: 1.51 ±0.25; SHAM EE: 2.14± 0.36; TRP EE: 2.08 ±0.23. (5MPT), SHAM SE: 0.37 ±0.07; TRP SE: 1.38 ±0.27; SHAM EE: 1.91± 0.20; TRP EE: 2.18 ±0.24. (6MPT), SHAM SE: 0.48 ±0.12; TRP SE: 1.53 ±0.20; SHAM EE: 2.12± 0.26; TRP EE: 2.24 ±0.27.<br>Two way ANOVA, Group: $F_{(3;208)}$ = 39.93; p<0.001; Time $F_{(7;208)}$ = 2.861; p=0.0072; interaction: $F_{(21;208)}$ =1.613; p=0.0486. Sidak's multiple comparisons test (main group effect); SHAM SE vs TRP SE, p<0.001; SHAM SE vs SHAM EE, p<0.001; SHAM SE vs TRP EE, p<0.001; TRP SE vs SHAM EE, p<0.01; TRP SE vs TRP EE, p<0.001; SHAM EE vs TRP EE, p=0.741 ns.<br>Sidak's multiple comparisons test (2WPT), SHAM SE vs TRP SE, p>0.05 ns; SHAM SE vs SHAM EE, p>0.05 ns; SHAM SE vs TRP EE, p>0.05 ns; TRP SE vs SHAM EE, p>0.05 ns; TRP SE vs TRP EE p>0.05 ns; SHAM EE vs TRP EE p>0.05 ns. (1MPT) SHAM SE vs TRP SE, p>0.05 ns; SHAM SE vs SHAM EE, p>0.05 ns; SHAM SE vs TRP EE, p>0.05 ns; TRP SE vs SHAM EE, p>0.05 ns; TRP SE vs TRP EE p>0.05 ns; SHAM EE vs TRP EE p>0.05 ns. (2MPT) SHAM SE vs TRP SE, p>0.05 ns; SHAM SE vs SHAM EE, p=0.008; SHAM SE vs TRP EE, p<0.001; TRP SE vs SHAM EE, p>0.05 ns; TRP SE vs TRP EE p>0.05 ns; SHAM EE vs TRP EE p>0.05 ns. (3MPT), SHAM SE vs TRP SE, p>0.05 ns; SHAM SE vs SHAM EE, p=0.002; SHAM SE vs TRP EE, p<0.001; TRP SE vs SHAM EE, p>0.05 ns; TRP SE vs TRP EE p>0.05 ns; SHAM EE vs TRP EE p>0.05 ns. (4MPT) SHAM SE vs TRP SE, p=0.033; SHAM SE vs SHAM EE, p<0.001; SHAM SE vs TRP EE, p<0.001; TRP SE vs SHAM EE, p>0.05 ns; TRP SE vs TRP EE p>0.05 ns; SHAM EE vs TRP EE p>0.05 ns. (5MPT) SHAM SE vs TRP SE, p=0.025; SHAM SE vs SHAM EE, p<0.001; SHAM SE vs TRP EE, p<0.001; TRP SE vs SHAM EE, p>0.05 ns; TRP SE vs TRP EE p>0.05 ns; SHAM EE vs TRP EE p>0.05 ns; (6MPT) SHAM SE vs TRP SE, p=0.017; SHAM SE vs SHAM EE, p<0.001; SHAM SE vs TRP EE, p<0.001; TRP SE vs SHAM EE, p>0.05 ns; TRP SE vs TRP EE p>0.05 ns; SHAM EE vs TRP EE p>0.05 ns.<br>Linear regression, SHAM SE: y= -0.02568x + 0.9651; Slope $F_{(1;54)}$ =35.31, deviation from zero: p<0.0001, Slope confidence intervals: -0,03434 to -0,01701; TRP SE: y= 0.01252x + 1.241; Slope $F_{(1;54)}$ = 1.626, deviation from zero: p=0.2078, Slope confidence intervals: -0,007170 to 0,03222; SHAM EE: y= 0.03737x + 1.337; Slope $F_{(1;54)}$ =10.77; deviation from zero: p=0.0018, Slope confidence intervals: 0,01454 to 0,06020; TRP EE: y= 0.03953x + 1.463; Slope $F_{(1;70)}$=10.97; deviation from zero: p=0.0015, Slope confidence intervals: 0,01572 to 0,06334.<br>N= 7 SHAM SE; 7 TRP EE; 7 SHAM SE; 9 TRP EE.

		Muscular fiber dimensions. **Figure 6C** Contralesional Gastrocnemius: Area: SHAM SE, 3136.14 ± 299.6µm^2, SHAM EE, 5974.27 ± 658.9µm^2, TRP SE, 6069.02 ± 278.2µm^2, TRP EE, 5144.89 ± 662.5µm^2. One way ANOVA, $F_{(3;8)}$=0.8217; p=0.5176 ns. Feret Min: SHAM SE, 71.20 ± 0.54µm, SHAM EE, 73.14 ± 4.4µm, TRP SE, 70.87 ± 1.9µm, TRP EE, 65.99 ± 3.2µm. One way ANOVA, $F_{(3;8)}$=1.092; p=0.4065 ns. N=3 SHAM SE; 3 SHAM EE; 3 TRP SE; 3 TRP EE.<br>Ipsilesional Gastrocnemius: Area: SHAM SE, 5123.43 ± 144.2µm^2, SHAM EE, 5904.83 ± 354.5µm^2, TRP SE, 5799.69 ± 753.1µm^2, TRP EE, 5013.47 ± 664. 1µm^2.One way ANOVA, $F_{(3;8)}$=0.7228; p=0.5661 ns. Feret Min: SHAM SE, 66.75 ± 1.5µm, SHAM EE, 72.61 ± 1.3µm, TRP SE, 70.59 ± 3.7µm, TRP EE, 63.66 ± 3.7µm. One way ANOVA, $F_{(3;8)}$=2.026; p=0.1888 ns. N=3 SHAM SE; 3 SHAM EE; 3 TRP SE; 3 TRP EE.<br>**Figure 6D** Contralesional Triceps: Area: SHAM SE, 7718.74 ± 348.6µm^2, SHAM EE, 7995.25 ± 814.8µm^2, TRP SE, 8021.259.5 ± 259.5µm^2, TRP EE, 7400.49 ± 201. 2µm2.One way ANOVA, $F_{(3;8)}$=0.3533; p=0.7882 ns. Feret Min: SHAM SE, 82.96 ± 2.7µm, SHAM EE, 81.17 ± 2.1µm, TRP SE, 83.03 ± 1.7µm, TRP EE, 80.07 ± 2.5µm. One way ANOVA, $F_{(3;8)}$=0.4026; p=0.7552 ns. N=3 SHAM SE; 3 SHAM EE; 3 TRP SE; 3 TRP EE.<br>Ipsilesional Triceps: Area: SHAM SE, 6226.07 ± 47.9µm^2, SHAM EE, 7731.97 ± 781.5µm^2, TRP SE, 6071.81 ± 901.3µm^2, TRP EE, 7300.12 ± 984. 6µm^2.One way ANOVA, $F_{(3;8)}$=1.072; p=0.4139 ns. Feret Min: SHAM SE, 74.75 ± 0.27µm, SHAM EE, 75.55 ± 3.6µm, TRP SE, 69.42 ± 6.3µm, TRP EE, 75.91 ± 3.9µm. One way ANOVA, $F_{(3;8)}$=0.5342; p=0.6716 ns. N=3 SHAM SE; 3 SHAM EE; 3 TRP SE; 3 TRP EE.
Figure 7	Cellular changes associated with EE	**Figure 7E** BDNF intensity in ipsilesional Cortex: SE, 86.5% low, 13.5% medium, 0% high intensity. EE, 71.75% low, 27.25 medium, 1% high intensity. Chi-square test $df_{(28;06;2)}$, p<0.001. N=100 cells/animal; 4 rats SE; 4 rats EE.<br>**Figures 7J and 7M** IBA1+ signal intensity: Ipsilesional/grafted CPu: SHAM 2MPT, 35.29 ± 2.1; SHAM SE, 5.39% ± 0.5; SHAM EE, 2.96% ±0.6; TRP 2MPT, 21.40 ± 1; TRP SE, 2.96% ±0.5; TRP EE, 3.57% ±0.6. One way ANOVA SHAM group, $F_{(2, 10)}$=157.1, p<0.001.Bonferroni post hoc test, 2MPT vs 6MPT SE, p<0.001; 2MPT vs 6MPT EE, p<0.001. One way ANOVA TRP group, $F_{(2, 13)}$=330.0, p<0.001.Bonferroni post hoc test, 2MPT vs 6MPT SE, p<0.001; 2MPT vs 6MPT EE, p<0.001. One way ANOVA tot, $F_{(5,23)}$=176.0, p<0.001.Bonferroni post hoc test, 2MPT vs 6MPT SE, p<0.001; 2MPT vs 6MPT EE, p<0.001. N= 5 SHAM 2MPT, 4 SHAM 6MPT SE; 4 SHAM 6MPT EE; 5 TRP 2MPT; 5 TRP 6MPT SE; 6 TRP 6MPT EE.<br>Ipsilesional cortex: TRP SE, 5.781% ± 0.5; TRP EE, 3.56% ±0.3; Contralesional cortex: TRP SE, 5.86% ±0.2; TRP EE, 3.87% ±0.7. One way ANOVA, $F_{(3;8)}$=6.491; p=0.0155. Unpaired t-test, ipsilateral CTX SE vs EE, p=0.018. N=3 rats SE, 3 rats EE

Additional Table 1. Statistical analysis: details related to all figures.

Antigen	Type	IHC	Manufacturer	Cat. Num.
BDNF	Rb	1:500	GeneTex	GTX132621
BDNF [3C11]*	Ms	1:500	Abcam	ab203573
Calbindin	Rb	1:500	Swant	CB38
Calretinin	Rb	1:500	Swant	7697
ChAT	Rb	1:400	Merck, Millipore	AB143
CTIP2	Rat	1:500	Abcam	Ab18465
DRD1	Rt	1:200	Sigma Aldrich	D2944
DRD2	Rb	1:200	Merck, Millipore	AB5084P
DARPP32	Rb	1:500	Abcam	Ab40801
ENK (ppENK)	Rb	1:400	Neuromics	RA14124
GABA	Rb	1:200	Merck, Millipore	MAB316
GFAP	Rb	1:500	Chemicon	AB5804
GFP	Chk	1:1000	AvesLabs	AB_2307313
GSX2	Rb	1:500	Merck, Millipore	ABN162
hNCAM	Ms	1:200	Santa Cruz Biotech.	Sc-106
HuNu	Ms	1:1000	Merck, Millipore	MAB128
IBA1	Rb	1:1000	Wako	019-19741
Ki67	Rb	1:1000	Abcam	Ab15580
muOR	Rb	1:1000	Abcam	AB10275-50
NESTIN	Ms	1:500	Merck, Millipore	ABD69
Parvalbumin	Rb	1:1500	Swant	PV25
RFP	Rb	1:1500	Rockland	600-401-379
SATB2	Ms	1:500	Abcam	Ab51502
STEM121	Ms	1:400	Takara Bio	Y40410
SUBP	Rb	1:400	CRB	CA-08-335
TBR1	Rb	1:500	Merck, Millipore	AB10554
TH	Rb	1:1000	IBJB	208020234
TH	Chk	1:1000	Merck, Millipore	AB9702

vGlut-1	Rb	1:1000	Synaptic Systems	135 303
vGlut-2	Gp	1:1000	Synaptic Systems	135 404

Additional Table2. List of primary antibodies and relative dilution factors used in the experimental procedures. Rb, rabbit; Ms, mouse; Chk, chicken; Gp, guinea pig.
* used to confirm BDNF pattern, as per Wosnitzka et al., 2020; doi: 10.1523/ENEURO.0462-19.2019.
